# Mannose-doped metal-organic frameworks induce tumor cell pyroptosis via the PERK pathway

**DOI:** 10.1186/s12951-023-02175-9

**Published:** 2023-11-15

**Authors:** Nianqiang Jin, Binhang Wang, Xinyao Liu, Chengcheng Yin, Xing Li, Zilin Wang, Xi Chen, Yunling Liu, Wenhuan Bu, Hongchen Sun

**Affiliations:** 1 Department of Oral Pathology, School and Hospital of Stomatology, China Medical University, Shenyang, 110001 P. R. China; 2 State Key Laboratory of Inorganic Synthesis and Preparative Chemistry, College of Chemistry, Jilin University, Changchun, 130012 P. R. China; 3 Sinochem Holdings Corporation Ltd., Beijing, 100031 P. R. China; 4 Sinochem Quanzhou Petrochemical Co., Ltd., Quanzhou, 362103 P. R. China; 5 Department of Center Laboratory, School of Stomatology, China Medical University, Shenyang, 110001 P. R. China; 6 Present Address: Liaoning Provincial Key Laboratory of Oral Diseases, Shenyang, 110001 P. R. China; 7 Hospital of Stomatology, Jilin University, Changchun, 130021 P. R. China; 8 Department of Oral and Maxillofacial Surgery, School and Hospital of Stomatology, Jilin University, changchun, 130021 P. R. China; 9 Department of Oromaxillofacial-Head & Neck Oncology, Shanghai Ninth People’s Hospital, Shanghai Jiao Tong University School of Medicine, College of Stomatology, National Center for Stomatology, National Clinical Research Center for Oral Diseases, Shanghai Key Laboratory of Stomatology, Shanghai Research Institute of Stomatology, Shanghai Jiao Tong University, Shanghai, 200011 P. R. China

**Keywords:** Immunotherapy, ER stress, GSDMD, Pyroptosis, Anti-tumor

## Abstract

**Background:**

The implementation of pyroptosis exhibits significant potential as a tactic to enhance tumor immune microenvironments. Previous applications of pyroptosis inducers have encountered various limitations, such as the development of drug resistance, manifestation of toxic side effects, and a deficiency in targeting capabilities. As a result, there is a growing demand for tumor therapeutic molecules that can overcome these obstacles. Therefore, the objective of this study is to develop a multifunctional nanospheres that addresses these challenges by enabling high-precision targeting of tumor cells and inducing effective pyroptosis.

**Results:**

We prepared a mannose-modified MOF called mannose-doped Fe_3_O_4_@NH_2_-MIL-100 (M-FNM). M-FNM could enter CAL27 cells through MR-mediated endocytosis, which caused in a significant increase in the level of intracellular ROS. This increase subsequently triggered ER stress and activated the PERK-eIF2α-ATF4-CHOP signaling pathway. CHOP then mediated the downstream cascade of Caspase-1, inducing pyroptosis. In in vivo experiments, M-FNM demonstrated excellent targeting ability and exhibited anti-tumor effects. Additionally, M-FNM reshaped the immune microenvironment by promoting the infiltration of anti-tumor immune cells, primarily T lymphocytes.

**Conclusions:**

M-FNM significantly decreased tumor growth. This novel approach to induce pyroptosis in tumor cells using M-FNM may offer new avenues for the development of effective immunotherapies against cancer.

**Supplementary Information:**

The online version contains supplementary material available at 10.1186/s12951-023-02175-9.

## Introduction

One of the most promising cancer immunotherapy approaches involves identifying and eliminating tumor cells through the recruiting and activating of T lymphocytes [1]. However, due to the unique microenvironment of tumors, the infiltration of immune cells that can inhibit tumor progression is highly restricted [2]. The scientific community has broadly acknowledged this issue, leading to the creation of several techniques to improve the immune surveillance of tumors. These strategies include immune checkpoint inhibitor therapy, combined chemoimmunotherapy, and chimeric antigen receptor T cell immunotherapy [3]. Despite these advancements, several problems, such as the negative side effects and poor generalizability of these approaches, must be addressed [4]. Therefore, new therapeutic strategies are urgently needed.

Recent studies have demonstrated that pyroptosis can trigger anti-tumor immune responses through releasing sufficient danger-associated molecular patterns (DAMPs), which is a promising therapeutic approach against cancer [5]. As a form of programmed cell death, the pivotal execution mechanism of pyroptosis involves caspase inflammasome mediated cleavage behavior, which impels cell lysis [6]. The cleavage of Gasdermin D (GSDMD) into the gasdermin N domain (N-GSDMD) leads to the formation of pores on the cell membrane, causing cytosolic contents to be released that contain various inflammatory factors and antigens [7]. Antigen-presenting cells, such as dendritic cells (DCs) and macrophages that are stimulated by these contents, activate and mature T cells, thereby leading to crucial anticancer immune responses [8]. Hence, inducing pyroptosis in tumor cells can significantly impede the growth of solid tumors.

Considering the intrinsic relationship between ferric iron-induced production of ROS and pyroptotic cell death, ferric iron has attracted considerable attention in the field of tumor chemodynamic therapy (CDT) due to its excellent enzymatic activity [9, 10]. Furthermore, previous studies have demonstrated that dispersing active sites into the frameworks of porous scaffolds, such as metal organic frameworks (MOFs), can effectively to achieve ultrahigh activity, selectivity, and atom economy in heterogeneous catalysis. This branch of CDT research is rapidly developing [11]. Therefore, adding iron ions to the building blocks of MOFs is an ideal choice to more efficiently stimulate the immune system. The efficiency of transforming endogenous H_2_O_2_ into hydroxyl radical (• OH) is limited by the high activation energy of Fe^3+^ from the chemical kinetics perspective [12]. Thus, internalizing Fe_3_O_4_ nanopartcles into MOFs could improve the catalytic efficiency of H_2_O_2_ after release. Besides, targeting becomes more challenging and crucial for cancer that involve metastatic or occult tumors. Optimizing interactions between ligands and receptors is essential for increasing the targeting effectiveness of nano-carriers [13]. Mannose, as an excellent targeting agent, aggregates indirectly to the tumor site by binding to surface receptors of antigen-presenting cells (APCs) or TAMs [14]. Despite this, the specific impact of mannose directly targeting tumor cells has been largely neglected. However, for tumor cells exhibiting high levels of mannose receptor (MR) expression, the use of mannose-modified nanocarriers could significantly enhance delivery efficiency.

Herein, we prepared a mannose-modified MOF (Mannose-doped Fe_3_O_4_@NH_2_-MIL-100, M-FNM). M-FNM could enter CAL27 cells through MR-mediated endocytosis and caused a significant increase in the levels of intracellular reactive oxygen species (ROS). According to mechanistic investigations, M-FNM not only mediated ROS diffusion and caused endoplasmic reticulum stress (ER Stress), but also activated the PERK-eIF2α-ATF4-CHOP signaling pathway. Furthermore, CHOP mediated the pyroptosis cascade of Caspase-1, which boosted the expression of the pyroptosis executive molecule N-GSDMD. Notably, compared to the primary metal complex (Fe_3_O_4_@NH_**2**_-MIL-100, FNM), M-FNM demonstrated superior targeting and antitumor effectiveness. Additionally, M-FNM reshaped the immune microenvironment and promoted the infiltration of tumor suppressive immune cells dominated by T lymphocytes. All results indicate that M-FNM pyroptosis inducers with outstanding CDT performances can provide a new strategy for tumor immunotherapy.

## Materials and methods

### Chemicals and reagents

Ferric chloride hexahydrate (FeCl_3_·6H_2_O), 1,3,5-Bfienzenetricarboxylic acid (H_3_BTC), and C_2_H_5_OH were purchased from Aladdin (Shanghai, China). Z-YVAD-FMK was purchased from Selleck (Shanghai, China). Liproxstatin-1 was purchased from ApexBio (Houston, USA). Mannose and 4-Phenylbutyric acid (4-PBA) were purchased from Sigma (NY, USA). FITC was purchased from MCE (NJ, USA). The polyvinylidene difluoride (PVDF) membrane was purchased from Merck (Darmstadt, Germany). The BCA protein assay kit, DCFH-DA, Fluo-4am, LDH Cytotoxicity Assay Kit, RIPA lysis buffer, and H&E staining kit were purchased from Beyotime Biotech (Nantong, China). Antibodies against GSDMD, N-GSDMD, PERK, ATF4, and cleaved Caspase-1 were purchased from Abcam (Shanghai, China). ASC, IL-1β, CD8, CD4, CD11c, and Foxp3 were purchased from Santa Cruz (SC, USA). CHOP, CD206, and tubulin were purchased from Proteintech (Wuhan, China). EIF2α and p-eIF2α were obtained from CST (Beverly, MA, USA). The Annexin V/PI double stain kit and superenhanced chemiluminescence (ECL) detection reagent were purchased from Yeasen (Shanghai, China). TNF-α and IL-1β ELISA kits were purchased from ABclonal (Wuhan, China).

### Cell culture

Human and mouse oral squamous cell carcinoma cells (CAL27/SCC-7) and human immortalized keratinocytes (HaCaT) were purchased from ATCC, as indicated. All cells were cultured at 37 °C and 5% CO_2_ in DMEM/1640, supplemented with 10% fetal bovine serum, 100 U/mL penicillin and 100 µg/mL streptomycin.

### Preparation of Fe_3_O_4_

Fe_3_O_4_ nanoparticles were synthesized using a solvothermal reduction method. Typically, 0.13 g of FeCl_3_·6H_2_O in 8 mL ethylene glycol was stirred for 30 min at room temperature. Then 1.3 g of sodium acetate was added to the solution and stirred for another 1 h. The resulting solution was transferred into a Teflon tube, sealed, and heated for 24 h at 180 ^o^C. Finally, the resulting magnetic Fe_3_O_4_ nanoparticles were centrifuged and washed with water three times, then redispersed in 10 mL H_2_O for further use.

### Preparation of Fe_3_O_4_@NH_2_-MIL-100 (FNM)

Amino-modified H_3_BTC was synthesized according to a previous work [15]. First, 2.7 g of FeCl_3_·6H_2_O was dissolved in 1 L C_2_H_5_OH, and 2.5 g of amino-modified H_3_BTC (NH_2_-H_3_BTC) was also dissolved in 1 L C_2_H_5_OH. Then, 5 mL of above Fe_3_O_4_ suspension and 10 mL of FeCl_3_ solution sonicated for 30 min, then centrifuged without wash. 10 mL of NH_2_-H_3_BTC solution was added and heated for 30 min at 65 ^o^C. Then, the mixture was centrifuged and washed with ethanal. Repeat this procedure for 8 times, followed by air drying for further use.

### Preparation of Mannose-doped Fe_3_O_4_@NH_2_-MIL-100 (M-FNM)

Fifty milligrams mannose was dissolved in 50 mL Phosphate buffered saline. 5 mg Fe_3_O_4_@NH_2_-MIL-100 nanoparticles was added to 10 mL mannose solution, kept 24 h at room temperature. The final product was collected by centrifugation and washed by PBS, followed by air drying.

### Transmission electron microscopy (TEM) analysis

The samples were dispersed on a copper grid coated with a carbon film. The morphology and structure of the samples were determined by JEM-2100 TEM (JEOL, Tokyo, Japan) with an accelerated voltage of 200 kV. Analyze images using the Gatan Microscopy Suite software (Las Vegas, Nevada, USA).

### N_2_ adsorption analysis

N_2_ adsorptions isotherm was measured on Quantachrome Autosorb-IQ2 liquid nitrogen automatic volumetric analyzer Bath (77 K). Using ultra-high purity N_2_ for adsorption experiments. This BET surface area analysis was performed by plotting *x/v (1-x) vs. x, where x = P/P0* (P0 = 1 bar), where *v* is the volume temperature and pressure (STP) of nitrogen adsorbed per gram of M-FNM under standard conditions, with satisfactory correlation coefficients and positive C constants were observed. The slope (*[c-1]/v*_*m*_*c*) and y intercept (the linear region between the dashed line of *1/v*_*m*_*c*) provide the single-layer capacity *v*_*m*_, which is used to calculate the capacity from A = *v*_*m*_*σ*_*0*_*N*_*AV*_, where σ_0_ is Avogadro’s cross-sectional area of the adsorbate at liquid density (16.2 Å^2^ for nitrogen) and N_AV_.

### X-ray diffractometer (XRD) analysis

The XRD pattern was recorded on a powder X-ray diffractometer (XRD, Rigaku D-MAX 2500/PC) equipped with a rotating anode and Cu/Kα_1_ radiation source (λ = 1.5406 Å), with a step size of 0.01°. Use Mercury software to simulate the XRD pattern of Mannose, Fe_3_O_4_, and MIL-100 (Fe) from crystallography data.

### Fourier transform infrared (FT-IR) spectroscopy

For FT-IR analysis, 20 mg KBr was ground together with 0.5 mg of the sample, and then pressed at 10 MPa. The spectrum was recorded on an FT-IR spectrophotometer (Thermo Scientific Nicolet iN5, Waltham, MA, USA) with a wavelength range of 400–4000 cm^− 1^. All collected data was processed using OMNIC Specta (Thermo Scientific, Carlsbad, CA, USA).

### X-ray photoelectron spectroscopy (XPS) analysis

The photoelectron spectrometer uses Axis Ultra imaging photoelectrons for analysis. The spectrometer (Thermo Scientific K-Alpha, Waltham, MA, USA) is equipped with Al K α (1486.7 eV) Quartz monochromator light source. During measurement, all samples were passed through plastic tape. The carbon C1s signal with binding energy of 284.8 eV is corrected by setting graphite as reference. All collected data was processed using Avantage software.

### Energy dispersive X-ray (EDX) analysis

The elements distribution was characterized using a scanning transmission electron microscopy (STEM, JEM-2100 F) with EDX.

### Cell viability

Adjust the density of CAL27 cells in logarithmic growth phase to 1×10^5^ cells/mL, take a 96-well plate, add 100 µL of cell suspension to each well, and place it in an incubator to culture overnight. Configuration containing detection M-FNM at different concentrations of 10, 20, 40, 80, 100, 200, and 300 µg/mL. After the cells are completely adherent, aspirate the medium and replace with the prepared medium. At the same time, a medium control group (negative group) and a blank control group containing only medium without cells were set, with 6 replicate wells in each group. For inhibition experiments by Z-YVAD-FMK and Liproxstatin-1. Z-YVAD-FMK was stored at 2 mM in 10% DMSO solution. 1 µL was added to 100 µL medium in the 96-well plate. Liproxstatin-1 was stored as 10 mM solution in DMSO. After 1:10 dilution in EtOH, 1 µL was added to 100 µL medium in the 96-well plate. After 24 h, add 10 µL of CCK-8 reagent to each well, continue to incubate for 1 h, and record the absorbance (OD) value of the microplate reader at a wavelength of 450 nm.

### Measurements for the intracellular ROS and concentration of Ca^2+^

Cells were planted onto confocal dish and cultured overnight. After that, cells were incubated with M-FNM (concentration: 300 µg/mL) for 4 h, respectively. The ROS levels were measured using the DCFH-DA reagent. The cells were incubated with DCFH-DA at a final concentration of 10 µM in FBS-free DMEM for 30 min at 37 °C in the dark, washed three times with FBS-free DMEM and resuspended in ice-cold PBS for the observe of ROS using a confocal laser scanning microscopy. The intracellular Ca^2+^ concentration was measured by the Fluo-4 am probe, and washed by PBS twice for confocal microscopy observation.

### Electron spin resonance (ESR) detection

The cells were inoculated into culture dishes, and after M-FNM treatment, the cell supernatants from all groups were collected. Dimethyl-1-pyrroline-N-oxide (DMPO) was chosen as the capture agent for hydroxyl radicals (•OH), and the detection was carried out following the procedure.

### Cellular uptake pathways

To explore the uptake pathway and efficiency of M-FNM by CAL27 cells, the specific experimental steps are as follows: 1×10^4^ CAL27 cells/well were seeded in confocal dishes and incubated for 24 h. The experimental groups: control group, FNM group, M-FNM group and Mannan + M-FNM group (mannan pretreated cells for 1 h with the concentration of 80 µg/mL). The medium of FITC-labeled FNM/M-FNM (concentration of 100 µg/mL) were further incubated in the dark for 1 h. Wash the cells three times with PBS to remove excess FNM/M-FNM. Cells in each group were fixed with absolute ethanol for 10 min, and then the nuclei were labeled with DAPI. The fluorescence signal of the cells was observed with a laser confocal microscope and the fluorescence intensity was analyzed with ImageJ.

### Detection of M-FNM-induced pyroptosis MCSs

Plate cells into Nunclon Sphera well plates at a density of 1×10^6^/well; incubate plates at 37 °C and 5% CO_2_; monitor MCSs formation for up to 7 days; and the cultures were changed every 72 h, carefully remove 500 µL of medium from each well and supplement with 500 µL of fresh medium. The M-FNM (300 µg/mL) were incubated with MCSs 48 h, then through immunofluorescence staining for cleaved Caspase-1, N-GSDMD and DAPI. Finally, the fluorescence signals of the MCSs were observed using a confocal microscope.

### Western blot analysis

CAL27/SCC-7 cells (1×10^5^) were seeded into 6-well plate. 24 h after seeding the cells, M-FNM (300 µg/mL) were added into 3 wells of the plate. the concentration of 4-PBA is 5mM and pretreated cells 1 h. The working concentration of nigericin was 10 µg/mL, and the cells were treated simultaneously with the M-FNM group. The other wells without any treatment and were used for the control measurements. At the onset of cell lysis, cells were washed twice with PBS and frozen at -80 °C. The wells that were treated equally were pooled for western blot assay. Western blot was performed according to standard procedures. The protein amount was normalized to the entire protein content via the Bradford assay and the result was normalized to the untreated control.

### Pyroptosis assays

Take the well-grown cells and spread them evenly into the culture plates or dishes needed for each experiment. The details of each treatment are as follows: to detect cell morphology/IL-1β/LDH release, 4-PBA (5 mM) was added 1 h in advance and then M-FNM (300 µg/mL) was added for 24 h. The control group received no treatment. To examine cell morphology, annexin V/PI were added to the cell-culture medium before subjected to imaging on a confocal microscope. The TEM is utilized to observe the microscopic structural changes of CAL27 cells. The image data shown are representative of at least three randomly selected fields. For the IL-1β levels in the supernatant, CAL27 cells in logarithmic growth phase were evenly distributed in the 6-well plate, ensuring a cell density of about 1×10^5^/well. The cells were placed in a cell culture incubator and incubated for 24 h for subsequent assays. Membrane integrity was analyzed by detecting the activity of LDH released into cell culture supernatants using the LDH cytotoxicity assay kit according to the manufacturer’s protocol.

### Immunofluorescence staining

CAL27 cells were cultured as described in the western blot experiments. Cultured cells were initially fixed with 4% PFA (paraformaldehyde), permeabilized with 0.5% Triton X-100 and blocked with 1% BSA/PBST. Primary antibodies were subsequently applied and incubated at 4 °C for 24 h, and then rinsed with PBS three times. After the cells rinsing with PBS, they were incubated with secondary antibody at 37 °C for 1 h, rinsed with PBS three times, and then counterstained with DAPI (10 µg/mL) and imaged using a confocal microscope.

To detect the infiltration of immune cells, immunofluorescence was performed and tumor histology was assessed according to the standard steps of tissue processing; then, the sections were further processed by immunofluorescence. The images were collected under a confocal microscope and the fluorescence intensity was analyzed using ImageJ.

### The biodistribution of nanoparticles in vivo

The procedures followed for daily animal care and experiments were performed under specific pathogen-free (SPF) conditions with an ambient temperature of 24 ± 2 °C, an air humidity of 40 − 70%, and a 12 h dark/12 h light cycle. BALB/c-nude mice (male, 5 weeks old) were purchased from SPF (Beijing) Biotechnology (Beijing, China). BALB/c-nude mice received a subcutaneous injection of 1 × 10^6^ CAL27 cells in the right flank to establish a solid tumor-bearing mouse model. After the tumor volume reached 200 mm^3^, the mice were intravenously administered Cy5.5-labeled FNM or M-FNM. Relying on the fluorescence of Cy5.5, a live-imaging system (PerkinElmer IVIS Spectrum, USA) was utilized to visualize the distribution of different agents in vivo. Eight hours after intravenous (i.v.) administration, the mice were sacrificed to collect major organs and tumors for in vivo imaging.

### M-FNM inhibited Tumor and activated antitumor immunity

BALB/c-nude mice received subcutaneous injection of 2 × 10^5^ CAL27 cells (a human oral squamous cell carcinoma cell line) at the right flank. 7 days post tumor inoculation, mice were randomly divided into 5 groups (5 mice per group), and received tail intravenous injection of PBS, mannose, Fe_3_O_4_, FNM, and M-FNM (Dosing frequency: every three days; Concentrations: 10 mg/kg). After that, tumor size and mouse weight were recorded for 12 days after tumor formation. Simultaneously, to assess the role of M-FNM-mediated anti-tumor immunity, a SCC-7 (a mouse oral squamous cell carcinoma cell line) tumor-bearing C57BL/6J mouse model was built and mice received the same treatments. The C57BL/6J mice (male, 5 weeks old) were purchased from SPF (Beijing) Biotechnology (Beijing, China). The infiltration of immune cells is detected according to the above immunofluorescence method. For cytokine analysis, serum samples were collected from pretreated mice and diluted before measurement. TNF-α and IL-1β were detected using ELISA kits according to the manufacturer’s protocol.

### Toxicological analysis

For the histological assessment, tumor-bearing C57BL/6J mice were killed after the indicated treatments, and the heart, liver, spleen, lung, and kidney were removed and fixed overnight with 4% formalin. After dehydration by gradient ethanol treatment, the tissue samples were embedded in paraffin and sectioned for hematoxylin and eosin staining. To verify the long-term biological safety of M-FNM, we administered a tail vein injection every three days to C57BL/6J mice for a total of 42 days. The injections included M-FNM and other groups (10 mg/mL). Collecting plasma from all groups for testing indicators such as creatinine (Cre), creatinine kinase (CK), lactic acid dehydrogenase (LDH), and aspartic acid transaminase (AST).

### Statistical analysis

Statistical data were expressed as means ± standard deviation (S.D.). Comparisons between different groups were carried out with Student’s t-test and analysis of variance (ANOVA) as appropriate. Values of *P* < 0.05 were considered to be statistically significant.

## Results

### Preparation and characterization of M-FNM

**Fig. 1:**
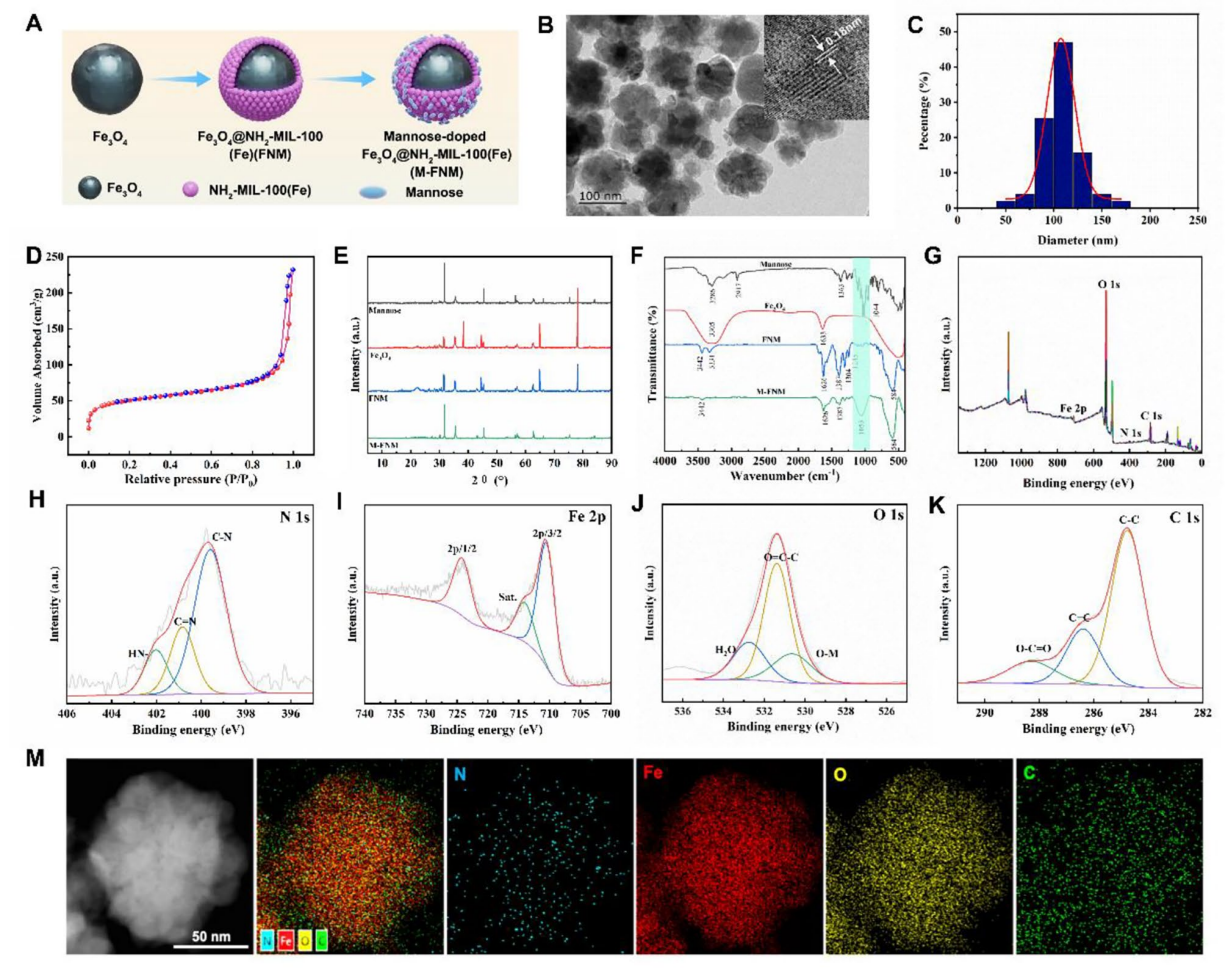
Preparation and Characterization of M-FNM. (**A**) Synthesis route. (**B**) TEM image of M-FNM. Inset: HRTEM. (**C**) Size distribution of M-FNM. (**D**) Nitrogen adsorption-desorption isotherms of M-FNM. (**E**) XRD pattern of Mannose, Fe_3_O_4_, FNM, and M-FNM. (**F**) FT-IR spectrum. (**G**-**K**) XPS spectra of wide-scan, N 1s Fe 2p, O 1s and C 1s of M-FNM. (M) Dark-field STEM image and EDX elemental mapping of M-FNM nano structures

Figure 1 A illustrates a schematic representation of the process to create the biocompatible and targeted iron delivery system, M-FNM. First, solvothermal reduction was utilized to create the Fe_3_O_4_ cores, followed by repeated coating procedures to produce FNM nanoparticles [16]. Mannose was then modified and applied to the surface of FNM to improve tumor targeting and the effectiveness of transmembrane administration. TEM was used to clarify the morphology of the produced structure, and the results showed that the Fe_3_O_4_, FNM, and M-FNM structures were spherical (Fig. 1B, Figure S1), with average diameters of approximately 85, 95, and 110 nm, respectively. The thickness of the NH_2_-MIL-100 layer was approximately 10 nm (Fig. 1C, Figure S2). Additionally, due to mannose modification, M-FNM exhibited a slightly larger size. High-resolution TEM (HRTEM) imaging confirmed the crystalline structure of M-FNM, revealing a lattice fringe of 0.18 nm (Fig. 1B). The BET surface area measurement indicated that M-FNM exhibited a porosity of 174.6 m^2^/g, which was favorable for the exchange and diffusion of ROS (Fig. 1D). Further investigation revealed that the pore size was approximately 9.3 nm, demonstrating the potential for facilitating anticancer medication transport (Figure S3). In aqueous solution, the zeta potential of M-FNM was found to be -23.3 mV (Figure S4). Moreover, X-ray diffraction (XRD) analysis confirmed the crystal structure of M-FNM, and all M-FNM nanoparticle diffraction peaks corresponded to the standard data of mannose, Fe_3_O_4_ (JCPDS file 19–0629, magnetite), and NH_2_-MIL-100 (Fig. 1E).

To gain further insight into M-FNM and additional structural information, Fourier transform infrared (FT-IR) analysis was conducted, and a distinct peak was observed at 1000 cm^− 1^ for M-FNM; this peak corresponded to mannose and was not present in the FNM spectrum. This result indicated that the mannose modification of FNM was successful (Fig. 1F). The successful synthesis of M-FNM was further validated by XPS spectrum, which showed the presence of N, Fe, O, and C (Fig. 1G). The N 1 s spectrum exhibited three peaks, in which the dominant peak was the C-N bond at 399.87 eV and the other two peaks represented the C = N bond at 401.4 eV and the NH- bond at 402.1 eV (Fig. 1H). The high-resolution spectra of Fe 2p displayed two subpeaks at 724.48 and 710.4 eV, which were assigned to Fe (III) 2p1/2 and Fe (II) 2p3/2, respectively (Fig. 1I). The asymmetric O 1 s spectrum displayed three peaks at 532.4, 531.4, and 530.6 eV, indicating the presence of -NO, O = C-C, and -OH, respectively (Fig. 1J). Furthermore, the C 1 s spectrum was deconvoluted into three peaks at 284.8 eV, 286.5 eV, and 288.8 eV, corresponding to C-C, C = C, and O-C = O, respectively (Fig. 1K). The X-ray energy spectrum mapping profile also revealed that N, Fe, O, and C were homogeneously distributed, which further supported that M-FNM was synthesized (Fig. 1M).

### Cellular uptake and cytotoxicity effects of M-FNM

**Fig. 2:**
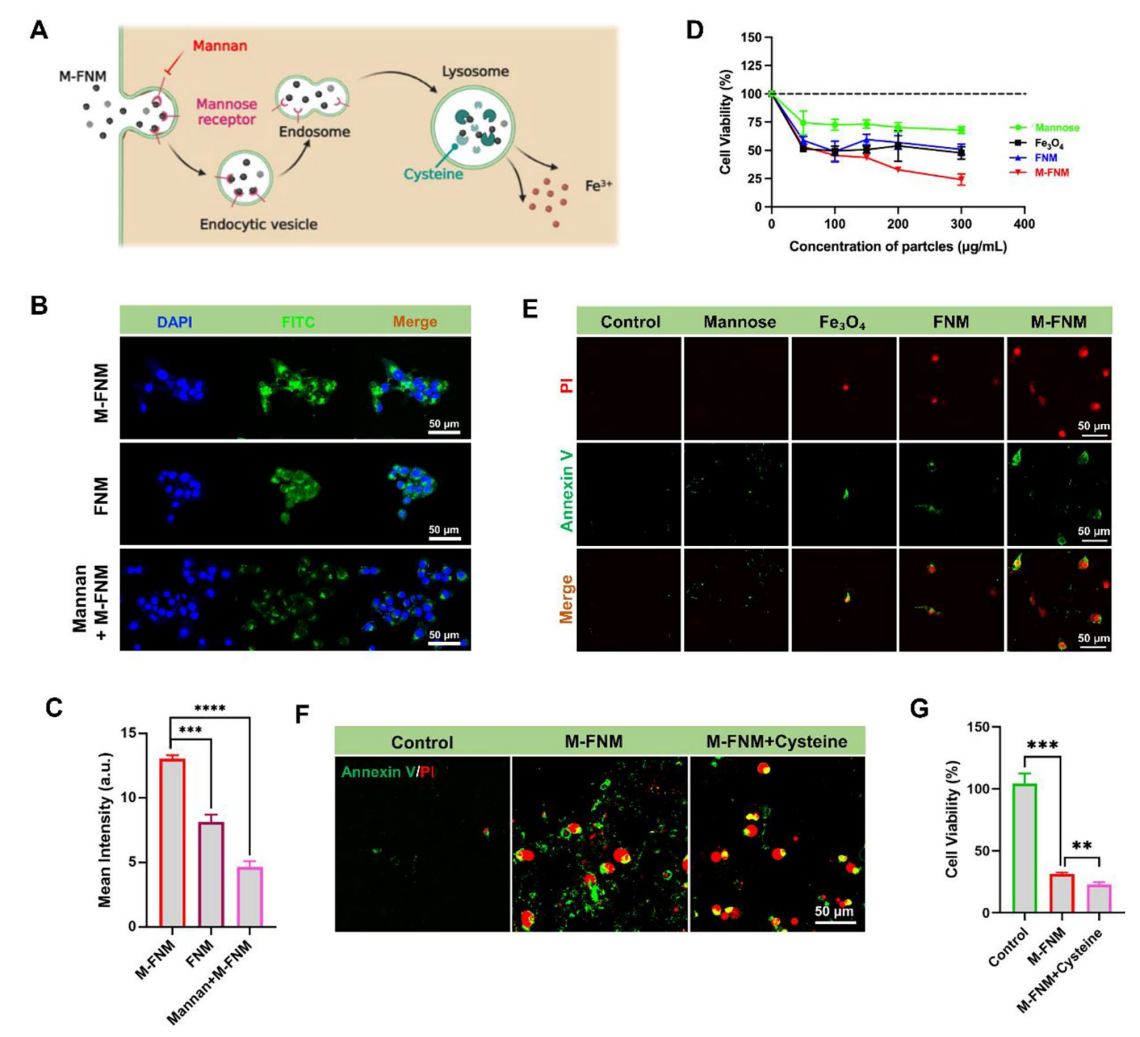
Cell uptake behavior and targeting effects of M-FNM. (**A**) Schematic illustration of cell uptake behavior and degradation process of M-FNM. (**B**) CLSM images of FNM/M-FNM (100 µg/mL) or with/without mannan labeled with FITC toward CAL27 cells. (**C**) Corresponding semi-quantitative analysis of FITC. (**D**) Relative cell viabilities of CAL27 cells after various treatments (300 µg/mL). (**E**) CLSM images of Annexin V/PI co-stained CAL27 cells after different treatments. (**F**) CLSM images of Annexin V/PI toward CAL27 cells with/without cysteine (10 µM). (**G**) Cytotoxicity of M-FNM toward CAL27 cells with/without cysteine (10 µM). (Mannan: 80 µg/mL; M-FNM: 300 µg/mL). Compared to control, *P < 0.05, **P < 0.01, ***P < 0.001, and ****P < 0.001. The mean values and standard deviations represent the average of biological triplicates

The cellular uptake of M-FNM is the most necessary process needed for its anticancer effect. Therefore, in this study, we investigated the uptake behavior of CAL27 cells by monitoring the fluorescence intensity of FITC-labeled nanoparticles (Fig. 2A). After CAL27 cells were incubated with FITC-labeled M-FNM for 1 h, we observed bright green fluorescence in the cytoplasm, indicating that CAL27 cells internalized M-FNM easily (Fig. 2B). We compared the cellular uptake efficiency of FNM and M-FNM and found that M-FNM exhibited a stronger fluorescence intensity, indicating that modification with mannose increased the efficiency of transmembrane transport (Fig. 2B, C). To clarify the role of MR in increasing transmembrane transport efficiency, we pretreated cells with mannan, an inhibitor of MR, to reduce the binding of mannose to MR [16, 17]. Pretreatment with mannan significantly decreased the efficiency of M-FNM cellular uptake (Fig. 2B, C). Simultaneously, the inhibitory effect of M-FNM on cell viability was significantly impacted by mannan intervention (Figure S5).

When evaluating antitumor effects in vitro, the inhibitory effect on cell proliferation activity is a key factor. Following treatment with mannose, Fe_3_O_4_, FNM, and M-FNM, the viability of CAL27 cells was reduced to 67.89%, 59.34%, 57.08%, and 32.84%, respectively (Fig. 2D). According to the results of the CCK-8 assay, M-FNM demonstrated an extraordinary killing power (IC_50_ ≈ 80 µg/mL) and the killing effect was concentration dependent (Figure S6). Given the positive anti-proliferative impact, Annexin V/PI was initially used to verify the antineoplastic effect. Compared to the other groups, the number of fluorescent cells stained with Annexin V/PI in the M-FNM group increased more significantly (Fig. 2E). Conditional responsive MOF degradation is also a vital feature in achieving targeted ion delivery. MIL-100 is broken down by the reductive action of abundant cysteine in lysosomes. [18] Therefore, we experimentally added cysteine to clarify the impact of MIL-100 degradation on the inhibitory effect of M-FNM. In further Annexin V/PI staining analyses, M-FNM caused cells to exhibit a vacuolar death pattern, which is not a characteristic of apoptotic cells. Subsequently, the formation of bubble-like cells in the M-FNM + cysteine group was clearly promoted by cysteine treatment (Fig. 2F). Moreover, the CCK-8 results showed that the addition of cysteine further inhibited cell proliferation (Fig. 2G), and a similar trend was observed as Annexin V/PI staining experiments. These results indicated that cysteine is involved in M-FNM degradation and helps M-FNM inhibit cell proliferation. Together, these results show that M-FNM relied on MR-mediated endocytosis to enter cells, then, M-FNM was further degraded and subsequently mediated cell death through the action of cysteine in lysosomes.

### M-FNM induced GSDMD-mediated pyroptosis in CAL27 cells

**Fig. 3:**
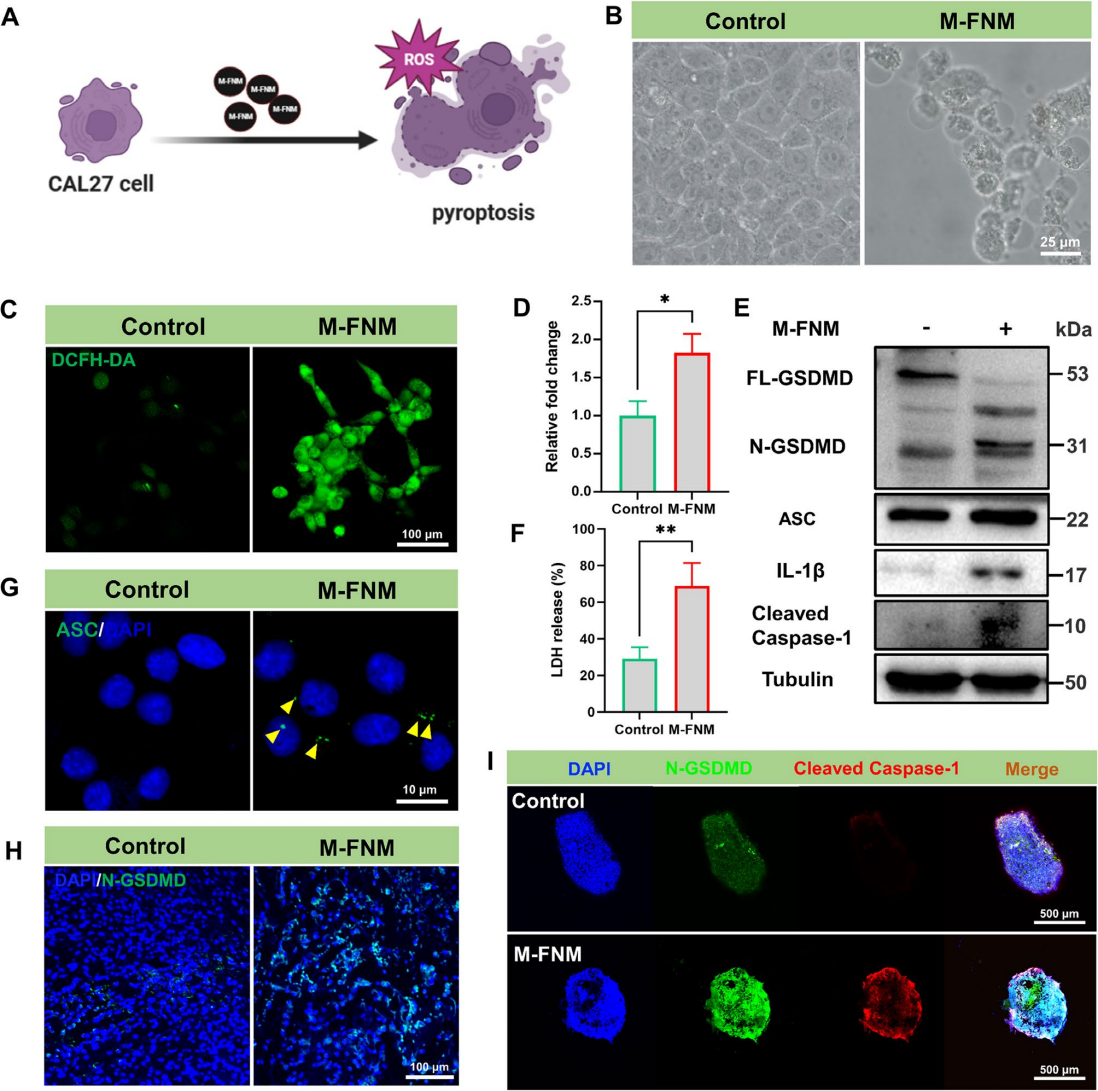
M-FNM induces GSDMD-mediated pyroptosis in CAL27 cells. (**A**) Schematic illustration of M-FNM Trigger pyroptosis. (**B**) Representative bright-field microscopy image of CAL27 cells with M-FNM treatment. (**C**) CLSM fluorescence images of ROS detected by the DCFH-DA after cells were incubated with M-FNM for 1 h. (**D**) Corresponding semi-quantitative analysis of DCFH-DA. (**E**) Western blot analysis of N-GSDMD ASC, IL-1β, and cleaved Caspase-1 in CAL27 cells were co-cultured with M-FNM, protein collection after vacuolar lysis of cells. (**F**) Release of LDH from the supernatant of CAL27 cells incubated with M-FNM for 24 h. (**G**) CLSM fluorescence images of ASC (yellow arrows) after cells were co-cultured with M-FNM. (**H**) CLSM fluorescence images of N-GSDMD in tumor after M-FNM treatment. (**I**) CLSM fluorescence images of MSCs after M-FNM treatment. (M-FNM: 300 µg/mL). Compared to control, *P < 0.05, **P < 0.01, and ***P < 0.001. The mean values and standard deviations represent the average of biological triplicates

The mode of CAL27 cell death mediated by M-FNM was investigated, and pyroptosis was identified as the likely mechanism due to the key characteristics observed, including the formation of transmembrane pores, which leads to cellular swelling and cytoplasmic vacuoles (Fig. 3A). Hence, cell morphology observation was initially introduced as the most intuitive method to compare treatment groups. The cells treated with M-FNM showed swelling characteristics and large bubbles, which was in sharp contrast to the morphology observed in the control group (Fig. 3B). Furthermore, TEM revealed the presence of vacuole-like structures in the cytoplasm and degradation of mitochondrial crests following M-FNM treatment (Figure S7). CDT can convert endogenous H_2_O_2_ into OH, which is the most harmful ROS because it triggers pyroptosis [19]. As a result, we investigated the level of intracellular ROS and discovered that they were considerably increased by M-FNM treatment (Fig. 3C, D). In addition, electron spin resonance (ESR) analysis confirmed that •OH was generated in the presence of M-FNM (Figure S8). Lactate dehydrogenase (LDH), an IL-1β inflammatory cytokine, is released from cells when pyroptosis occurs [20–22]. Similarly, following M-FNM treatment, the concentrations of LDH and IL-1β in the supernatant of CAL27 cells dramatically increased (Fig. 3F, S9). Furthermore, apoptosis-associated speck-like protein (ASC) forms macromolecular dimers in the cytoplasm during inflammasome activation when Caspase-1 separates from inflammasomes [23]. Immunofluorescence research revealed that M-FNM also promoted the formation of ASC specks (Fig. 3G). To investigate whether Caspase-1 was activated and subsequently cleaved GSDMD into N-GSDMD, western blot analysis was used to assess protein expression. As results shown, elevated expression of cleaved Caspase-1 and N-GSDMD proteins was observed in the M-FNM group, supporting findings that this treatment increases the permeabilization of the plasma membrane and the formation of membrane pores. The high ASC and cleaved Caspase-1 expression observed in the M-FNM group supports Caspase-1 activation. IL-1β, a Caspase-1 substrate, and increased expression of the mature IL-1β form (p17) are also indicative of Caspase-1 activation (Fig. 3E). In addition, a multicellular spheroids (MSCs) tumor model was established for phenotypic analysis of the antitumor activity of M-FNM using CAL27 cells. Compared to cells in culture, MSCs exhibit more pronounced associations with solid tumor [24]. Compared to the control group, CLSM images showed that M-FNM increased the expression of N-GSDMD and cleaved Caspase-1 in MSCs (Fig. 3I). However, the CCK-8 results demonstrated that cell viability was preserved when Z-YVAD-FMK (a caspase inhibitor) was used, further demonstrating that M-FNM induced pyroptosis (Fig. S10). Due to cell viability was not restored by treatment with liproxstatin-1 (a ferroptosis inhibitor) in M-FNM group, it indicates that cell death induced by M-FNM does not occur via ferroptosis (Figure S11). Simultaneously, the presence of M-FNM did not lead to changes in the level of MLKL phosphorylation, which is a marker of necroptosis. In addition, the protein expression levels of apoptosis-related markers, such as Bcl-2 and cleaved caspase-3, did not show significant changes (Figure S12). Moreover, following M-FNM treatment, the N-GSDMD expression level was highly elevated in CLSM images of tumor tissue (Fig. 3H). These findings also demonstrated the role of M-FNM in mediating pyroptosis and the predominance of the caspase family in pyroptosis. Overall, we concluded that M-FNM kills tumor cells by triggering pyroptosis.

### M-FNM activated the PERK pathway to induce pyroptosis

**Fig. 4:**
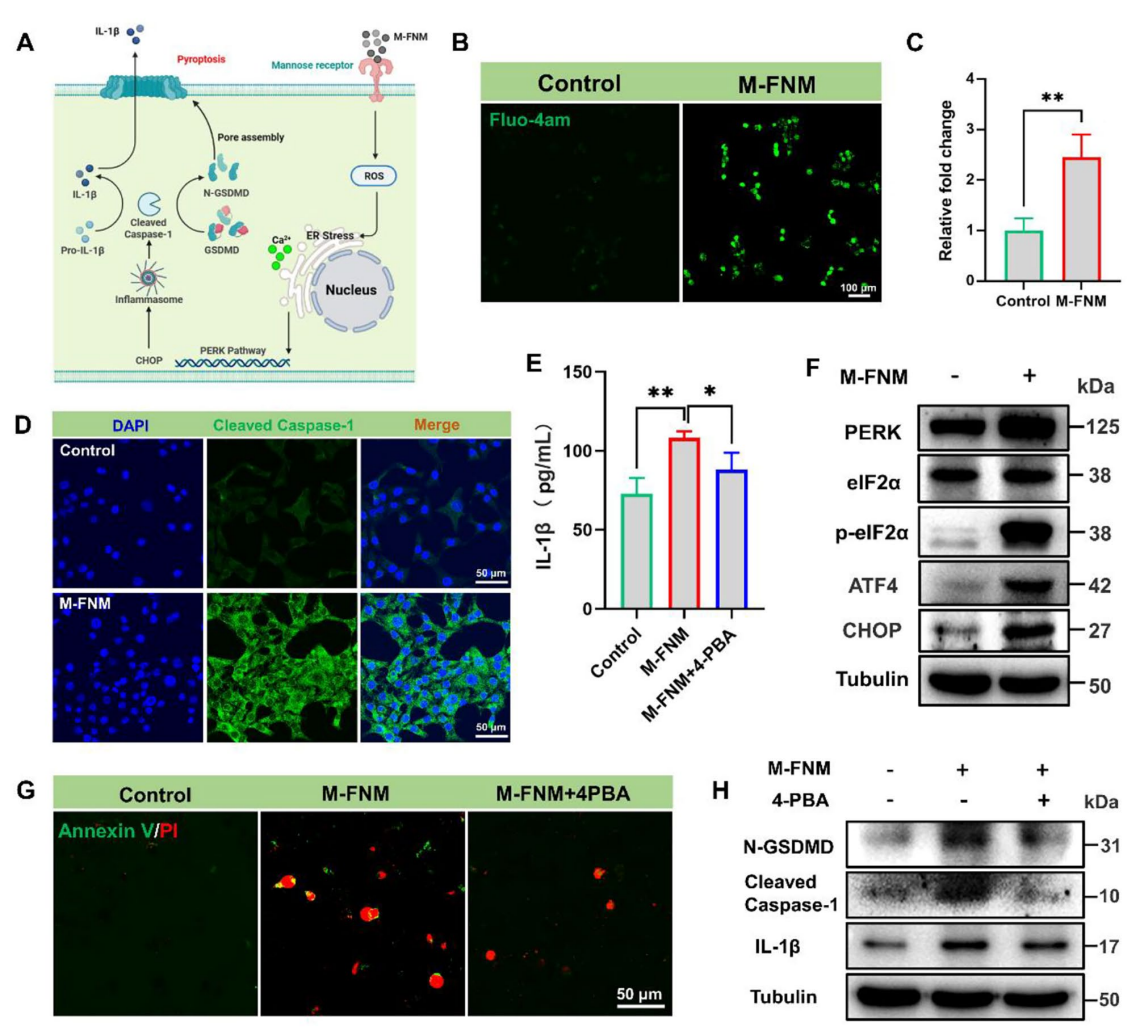
M-FNM activates the PERK pathway to induce pyroptosis. (**A**) Mechanism illustration of M-FNM mediates pyroptosis. (**B**) CLSM images of calcium ions in the cytoplasm after M-FNM treatment. (**C**) Corresponding semi-quantitative analysis of Fluo-4am. (**D**) CLSM fluorescence images of cleaved Caspase-1 in CAL27 cells after M-FNM treatment. (**E**) IL-1β release in supernatant after M-FNM treatment. (**F**) Western blot analysis PERK pathway in CAL27 cells after M-FNM treatment. (**G**) CLSM images of Annexin V/PI toward CAL27 cells. (**H**) Western blot analysis of N-GSDMD, cleaved Caspase-1, and IL-1β in CAL27 cells. (4-PBA: 5mM; M-FNM: 300 µg/mL). Compare to control, *P < 0.05, **P < 0.01, and ***P < 0.001. The mean values and standard deviations represent the average of biological triplicates

Although M-FNM treatment triggers pyroptosis, the upstream regulatory process mediating this process remains unknown. Therefore, the mechanism by which M-FNM causes pyroptosis was investigated (Fig. 4A). ROS are among the stimulators of ER stress, a cellular defense mechanism that maintains intracellular homeostasis [25]. Specifically, PERK is a key ER stress sensor that can sense intracellular ROS levels and mediate ER stress [26]. Among the cellular indicators, the leakage of calcium ions into the cytoplasm is a sign of ER stress [27, 28]. As expected, calcium ions in the cytoplasm increased dramatically in response to M-FNM (Fig. 4B, C). These results suggest that the cascade reaction of Caspase-1 and the occurrence of pyroptosis are mediated by ER stress [28–30].

Evaluating the expression levels of p-eIF2α and other PERK signaling pathway proteins is the gold standard for determining whether the PERK signaling pathway is activated [31]. Elevated expression of p-eIF2α, PERK, ATF4, and CHOP was observed in the M-FNM group, but the total expression of eIF2α was not altered (Fig. 4F). Thus, western blot results showed that the PERK signaling pathway was activated by M-FNM. In addition, increasing the expression of CHOP helps activate Caspase-1 [32]. Based on CLSM images, the green fluorescence signal in the M-FNM group confirmed that cleaved Caspase-1 expression was upregulated. (Fig. 4D). To investigate the role of the PERK pathway in Caspase-1 activation, 4-PBA, a small molecule inhibitor of the PERK pathway, was chosen [33, 34]. The expression of N-GSDMD, cleaved Caspase-1, and L-1β was verified through western blot analysis. Secreted IL-1β in the cell supernatant was also detected by ELISA. The 4-PBA-related group inhibited expression of the above proteins (Fig. 4H) and caused a substantial decrease in IL-1β secretion in the cell supernatant (Fig. 4E). Additionally, the results obtained from cell morphology investigations and Annexin V/PI staining revealed that 4-PBA interfered with pyroptosis caused by M-FNM (Fig. 4G). To summarize, M-FNM induced the activation of the PERK-eIF2α-ATF4-CHOP pathway in CAL27 cells, lading to cleaved Caspase-1 cleaves full-length GSDMD into N-GSDMD with membrane-pore forming activity and immature IL-1β into mature IL-1β. Therefore, ER stress and the PERK-eIF2α-ATF4-CHOP pathway play crucial roles in M-FNM-induced pyroptosis.

### Tumor targeting and inhibitory effect of M-FNM

The effective accumulation of drugs in the local tumor is essential for antitumor effects and does not damage normal tissue [35, 36]. To verify the targeting effect in vivo, FNM and M-FNM nanoparticles were labeled with Cy5.5 and injected into CAL27 tumor-bearing mice (BALB/c-nude) through the tail vein. Compared to FNM, M-FNM was more enriched in the tumor 6 h after injection, as determined by ISVS analysis. At 8 h after injection, significant fluorescence intensity could be detected in tumors in the M-FNM group (Figure S13A). These results indicated that M-FNM exhibited an excellent CAL27 tumor targeting effect. Additionally, the tumor and vital organs of the mice were collected after 8 h, and the fluorescence results showed that the M-FNM group had greater fluorescence aggregation in the tumor, which was consistent with the in vivo imaging results., The accumulation of M-FNM in the kidney suggests that the metabolism of M-FNM may be completed in the kidney compared to other organs (Figure S13B).

**Fig. 5:**
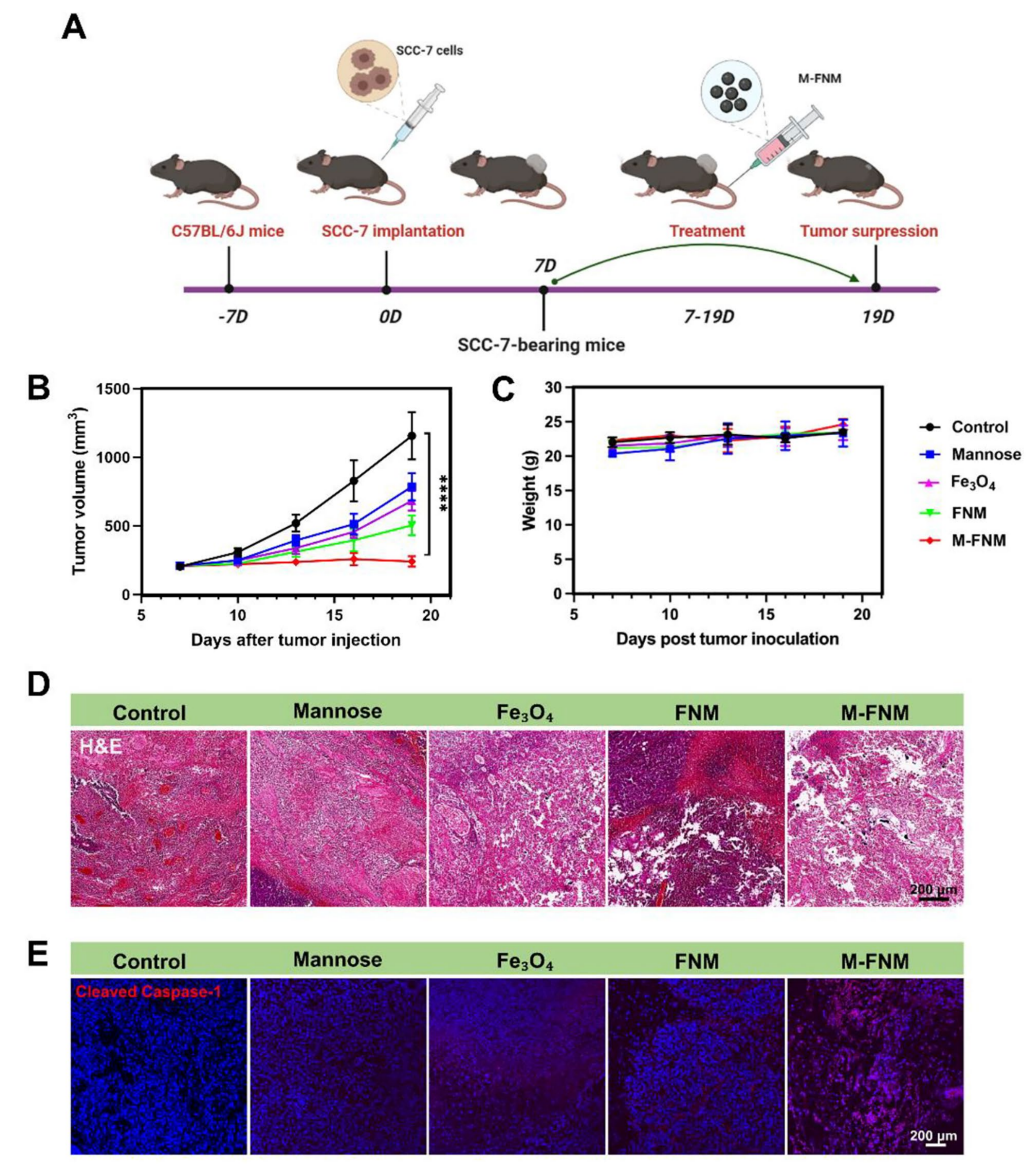
Antitumor activity of M-FNM against SCC-7 tumor xenografts in mice. (**A**) Treatment schedule of utilizing M-FNM for antitumor therapy. (**B**-**C**) Tumor volume and body weight of SCC-7-tumor-bearing mice with different treatments (C57BL/6J, n = 5). Mice received PBS, Mannose, Fe_3_O_4_, FNM, or M-FNM injection (10 mg/kg), respectively. (**D**) Histological images of SCC-7 tumors after different treatments. (**E**) CLSM fluorescence images of cleaved Caspase-1 in tumor after various treatments. Compared to control, *P < 0.05, **P < 0.01, ***P < 0.001, and ****P < 0.001

To evaluate the antitumor effect of M-FNM in vivo, we created a 5-week-old male BALB/c-nude mouse model of subcutaneous CAL27 tumors. Figure S14 displays the treatment strategy. The tumor volume was monitored and verified, which is an important indicator for the effectiveness of the treatment. After 12 days of treatment, the average tumor volume was 355 mm^3^. Compared to the tumor volume observed in other groups, the tumor volume in M-FNM group was much smaller (Figure S15). Digital photos of free tumors for each group are presented in Figure S16. As nude mice lack a sound immune system, the antitumor effect of M-FNM could not be fully evaluated. Therefore, to investigate the effectiveness of immunotherapy, a 5-week-old male C57BL/6J mouse model of SCC-7 subcutaneous tumors was developed (Fig. 5A). Following M-FNM treatment, SCC-7 cells also undergo pyroptosis (Figure S17). Tumor growth was significantly inhibited in the M-FNM therapy group. After 12 days of treatment, the typical tumor volume in mice was 242 mm^3^. Compared with the Fe_3_O_4_ group with mild tumor inhibition, the average volume of mice treated with M-FNM decreased by 441 mm^3^ (Fig. 5B, Figure S18), suggesting that M-FNM treatment notably inhibited the growth of SCC-7 tumors. For histopathological evaluation, hematoxylin and eosin (H&E) staining examination was performed to determine the degree of cell damage and necrosis. Normal morphology was only retained in the SCC-7 cells in the control and mannose groups, whereas the M-FNM group showed the most histological damage (Fig. 5D). Immunofluorescence analysis was also used to detect the expression of pyroptosis-related proteins further clarify the mechanism underlying cell death. After M-FNM treatment, the expression of cleaved Caspase-1 increased, indicating pyroptotic cell death (Fig. 5E). However, in the two in vivo models, M-FNM treatment did not result in a substantial change in mouse body weight (Fig. 5C, Figure S19).

### M-FNM boosted anti-tumor immunity in vivo

**Fig. 6:**
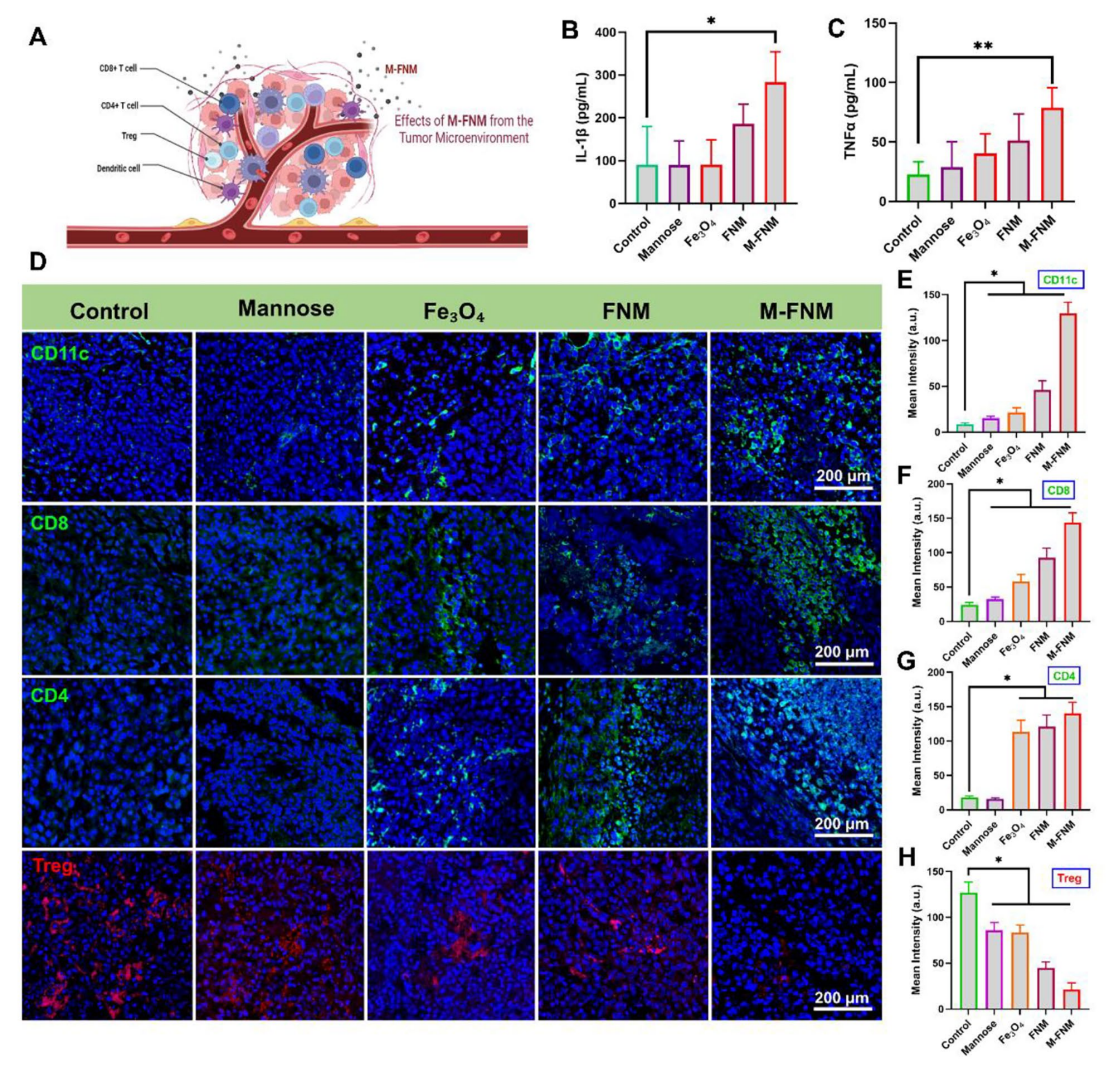
M-FNM boosted antitumor immunity. (**A**) Schematic illustration of immune cells infiltration. (**B**) IL-1β and (**C**) TNF-α level in mouse serum after treatments. (**D**) Infiltration of CD11c-labeled dendritic cells, CD8/CD4-labeled T lymphocytes, Foxp3-labeled Treg cells in tumor tissue. (**E**-**H**) Quantitative analysis of fluorescence intensity of various indicators. Compare to control, *P < 0.05, **P < 0.01, and ***P < 0.001

According to previous studies, pyroptosis is advantageous for triggering an anticancer response because it involves recruiting and activating immune cells [37] (Fig. 6A). DAMPs are released by tumor cells during pyroptosis, which causes an increase in the infiltration of mature dendritic cells (DCs) and further T-cell activation [37]. To examine the potential role of M-FNM-induced pyroptosis in the activation of antitumor immunity, DCs and T-cells infiltration were investigated by immunofluorescence staining. The findings showed that M-FNM could efficiently promote the infiltration of CD11c^+^ DCs and CD8^+^/CD4^+^ T cells (Fig. 6D, E, F, G). Mice treated with FNM displayed less effective induction of pyroptosis, which resulted in less effective CD11c^+^ DCs and CD8^+^/CD4^+^ T-cell infiltration. In addtion, the proportion of Treg cells in the M-FNM group was significantly lower than that in the other groups (Fig. 6D, H).

Furthermore, ELISA was utilized to quantitatively assess the concentration of cytokines in mouse serum. The serum of mice in the FNM and M-FNM groups displayed significantly higher levels of IL-1β and TNF-α than that of mice in the PBS, mannose, and Fe_3_O_4_ groups. Notably, compared to other therapies, M-FNM was more effective in eliciting an antitumor immune response in mice, as evidenced by the higher levels of cytokine production observed (Fig. 6B, C). Given that the biological toxicity of nanomaterials is crucial in translating their utility from experimental settings to practical applications, we conducted biocompatibility analysis in vivo using H&E staining. The main organs (including the heart, liver, spleen, lung, and kidney) were stained with H&E, and no substantial inflammatory lesions or pathological alterations were observed in any group, which supports the good biocompatibility of M-FNM (Figure S20). In addition, M-FNM did not cause any significant toxicity-related changes, including alterations in creatinine (Cre), creatinine kinase (CK), lactate dehydrogenase (LDH), and aspartate aminotransferase (AST) levels (Figure S21).

## Discussion

Transition metals, such as Fe, are the most common constituent elements of nanozymes and are reliable candidates for CDT of malignant tumors [38]. During the CDT process, endogenous H_2_O_2_ is transformed into • OH, and the Fe valence state changes from ferrous to trivalent iron [39]. Even though iron is a key candidate for CDT of tumors, the targeting effect on occult malignant tumors, such as oral squamous cell carcinoma, remains insufficient, and the impact is limited. Additionally, the efficiency of iron delivery is a scientific issue that requires special attention [40]. In this study, we successfully prepared a Fe_3_O_4_-containing MOF that is surface-modified with mannose. The mannose modification significantly improved targeting and utilized MR-mediated endocytosis to improve transmembrane transport efficiency and enhance therapeutic efficacy. This modification choice was based on the high local expression of MR in malignancies [13]. Moreover, mannose can disrupt the glycometabolism of tumor cells, resulting in slow tumor growth. Additionally, it boosts cell sensitivity to chemotherapy drugs [41], thereby creating a synergistic inhibitory effect with Fe^3+^ on tumor growth. In addition, MR is highly expressed in the CAL27/SCC-7 cell models used in this study. With more abundant iron ions, the catalysis of H_2_O_2_ will be sustained and efficient, thereby the continuous production of • OH is maintained and tumor regression is mediated.

After nanomaterials are internalized, they must enter lysosomes, and the unique lysosomal environment exhibits positive and negative effects on the functional properties of these materials [42, 43]. The M-FNM could cleverly respond to cysteine in lysosomes to achieve its own antitumor effect. Subsequently, a mechanism underlying M-FNM’s anticancer activity involves pyroptosis. Pyroptosis is distinguished by the activation of Caspase-1 and an increase in N-GSDMD expression [44]. However, the development of anticancer medications that target pyroptosis is severely constrained because the upstream mechanism through which pyroptosis occurs is not fully known. Our findings showed that ER stress and the PERK pathway are vital for M-FNM-mediated pyroptosis. Furthermore, other anticancer nanomaterials could regulate pyroptosis by targeting the PERK pathway.

The antitumor effect of M-FNM could’t been fully demonstrated in vivo in the BALB/c-nude tumor-bearing mouse model due to the lack of immune cells. M-FNM converts “cold” tumors into “hot” tumors by recruiting immune cells, prohibiting the growth of tumors in C57BL/6J tumor-bearing mice. Therefore, in future transformation therapies, M-FNM-induced pyroptosis may increase the susceptibility of tumors to immune checkpoint inhibitors. Additionally, we will further consider using treatment combinations with immune checkpoint inhibitors and investigate the synergistic effect of targeting magnetism and mannose to identify combinations with more accurate tumor targeting effects.

## Conclusions

In summary, we created a mannose-modified iron-based MOF that can easily be undergo endocytosis by cancer cells. Importantly, we show that lysosomal cysteine is a prominent mediator of M-FNM cytotoxicity. Additionally, M-FNM facilitated PERK-eIF2α-ATF4-CHOP signaling pathway activation, which subsequently stimulated the caspase-1 cascade, promoted N-GSDMD formation and ultimately resulted in pyroptosis. M-FNM activated the antitumor immune response and generated an excellent synergistic antitumor effect with directly induced pyroptosis. Our results further broaden the application prospects of the iron transition metal for CDT-based treatment, highlighting the great transformation value of M-FNM.

### Electronic supplementary material

Below is the link to the electronic supplementary material.

Supplementary Material 1

## Data Availability

The datasets used and/or analyzed during the current study available from the corresponding author on reasonable request.
