# Supplementary material for: Mannose-doped metal-organic frameworks induce tumor cell pyroptosis via the PERK pathway
Source: J Nanobiotechnology. 2023 Nov 15;21:426. doi: 10.1186/s12951-023-02175-9 (PMC10647064; doi:10.1186/s12951-023-02175-9)
Supplement: Supplementary file 1 — Supplementary Material 1 [file 12951_2023_2175_MOESM1_ESM.docx]

**Supporting Information**

**Mannose-Doped Metal-Organic Frameworks Induce Tumor Cell Pyroptosis Via the PERK Pathway**

Nianqiang Jin^a^, Binhang Wang^b^, Xinyao Liu^bhi^, Chengcheng Yin^cg^, Xing Li^d^, Zilin Wang^ef^, Xi Chen^a^, Yunling Liu^b^*, Wenhuan Bu^d^* and Hongchen Sun^d^

^a^ Department of Oral Pathology, School and Hospital of Stomatology, China Medical University, 110001, Shenyang, P. R. China

^b^ State Key Laboratory of Inorganic Synthesis and Preparative Chemistry, College of Chemistry, Jilin University, Changchun 130012, P. R. China

^c^ Department of Center Laboratory, School of Stomatology, China Medical University, Shenyang 110001, P. R. China

^d^ Hospital of Stomatology, Jilin University, Changchun, 130021, P. R. China

^e^ Department of Oral and Maxillofacial Surgery, School and Hospital of Stomatology, Jilin University, Changchun 130021, P. R. China

^f^ Department of Oromaxillofacial-Head & Neck Oncology, Shanghai Ninth People's Hospital, Shanghai Jiao Tong University School of Medicine, College of Stomatology, National Center for Stomatology, National Clinical Research Center for Oral Diseases, Shanghai Key Laboratory of Stomatology, Shanghai Research Institute of Stomatology, Shanghai Jiao Tong University, Shanghai 200011, P. R. China

^g^ Liaoning Provincial Key Laboratory of Oral Diseases, Shenyang 110001, China

^h^ Sinochem Holdings Corporation Ltd., Beijing 100031, P. R. China

^i^ Sinochem Quanzhou Petrochemical Co., Ltd., Quanzhou 362103, P. R. China

***3.Supplementary Figures***

***Supplementary Figure S1***

***
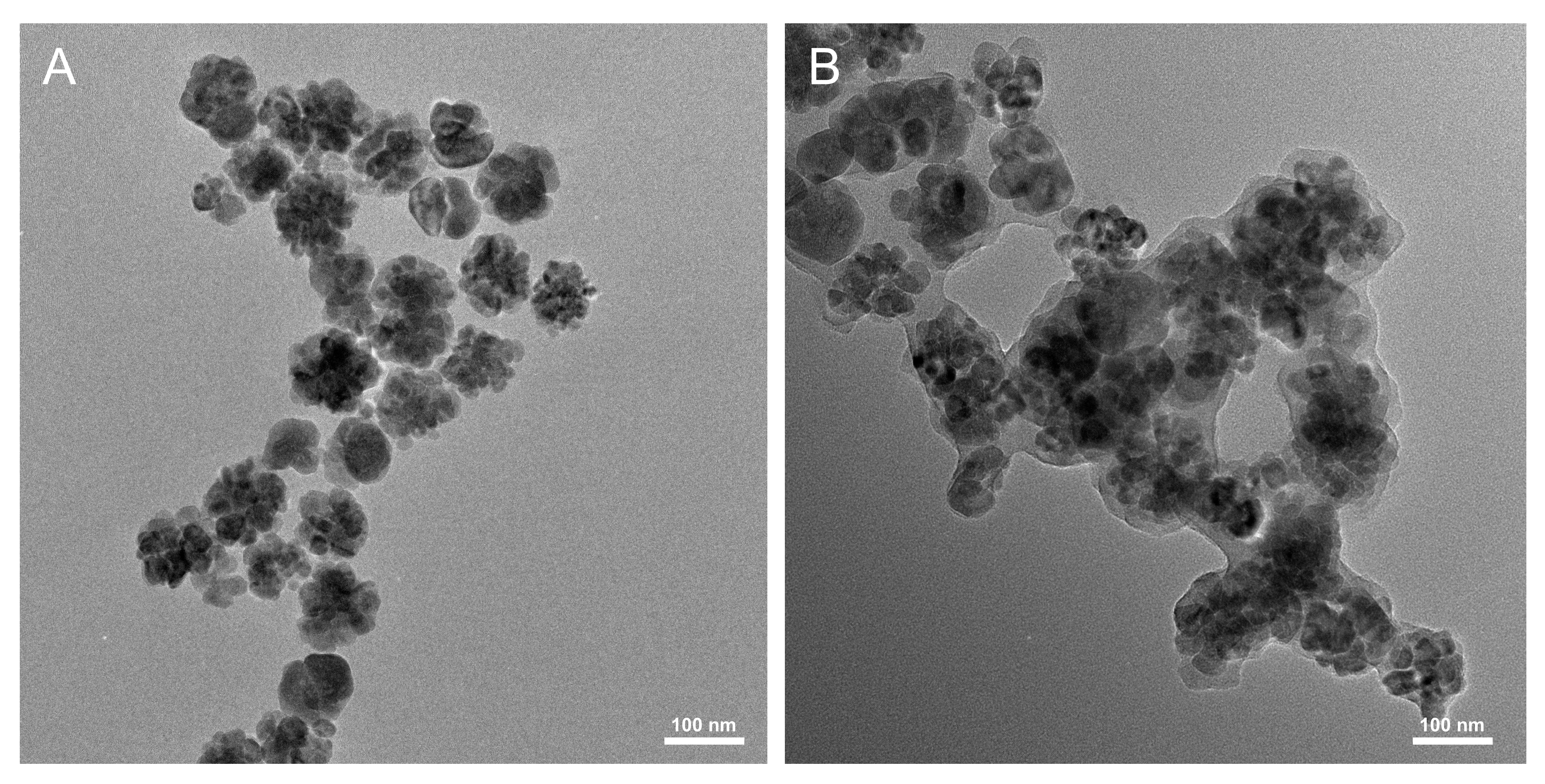
***

**Supplementary Figure S1. TEM images of the nanoparticles.** TEM of (A) Fe_3_O_4_ and (B) Fe_3_O_4_@NH_2_-MIL-100 (FNM) particles. The images show the homogenous shape and size of the nanoparticles, the morphology was maintained during NH_2_-MIL-100 coating.

***Supplementary Figure S2***

***
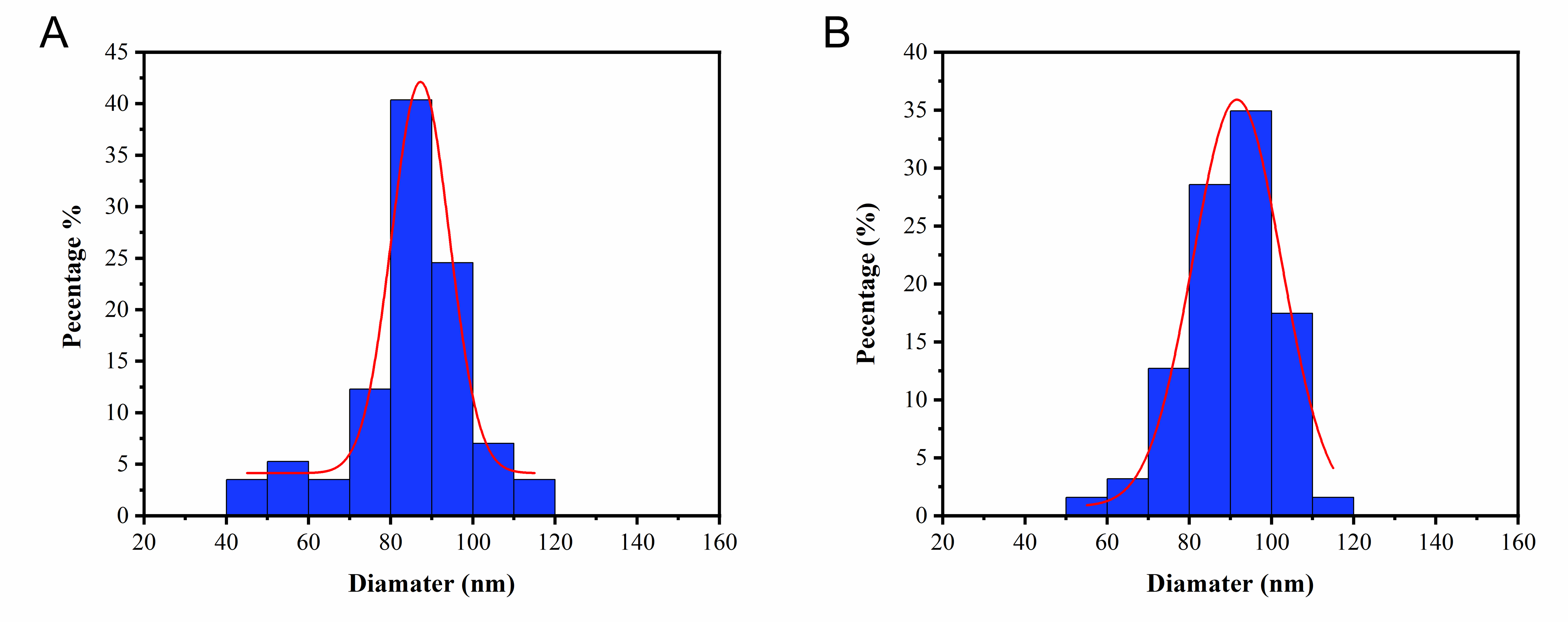
***

**Supplementary Figure S2. Hydrodynamic size distribution of particles.** Sizes of (A) Fe_3_O_4_ and (B) FNM nanoparticles.

***Supplementary Figure S3***

***

***

**Supplementary Figure S3.** The corresponding pore size distribution of M-FNM.

***Supplementary Figure S4***

**
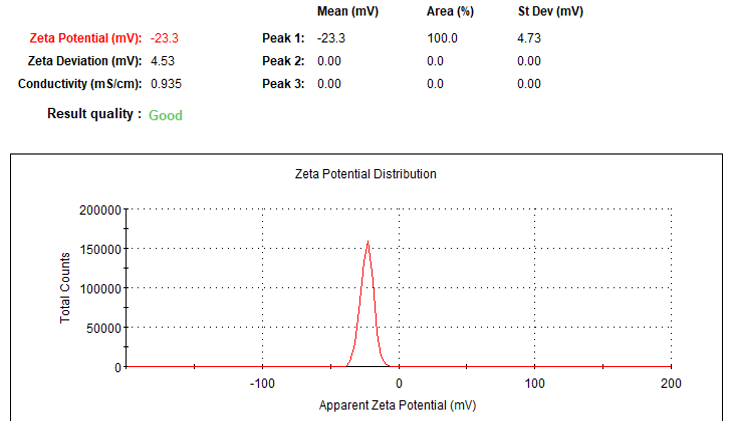
**

**Supplementary Figure S4.** Zeta potential of M-FNM.

***Supplementary Figure S5***


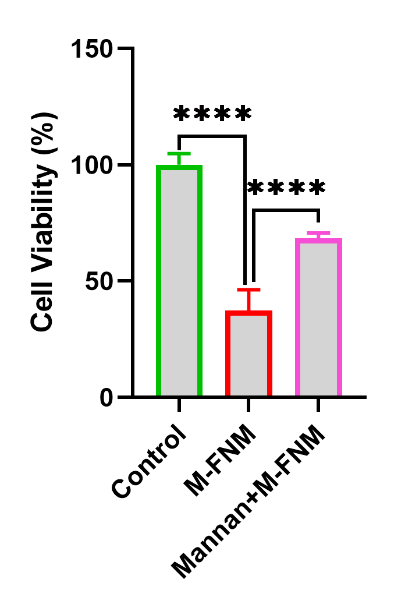


**Supplementary Figure S5.** Mannan decreases the inhibitory effect of M-FNM on cell viability. Compared to control or M-FNM, **P* < 0.05, ***P* < 0.01, ****P* < 0.001, and *****P* < 0.0001.

***Supplementary Figure S6***

**
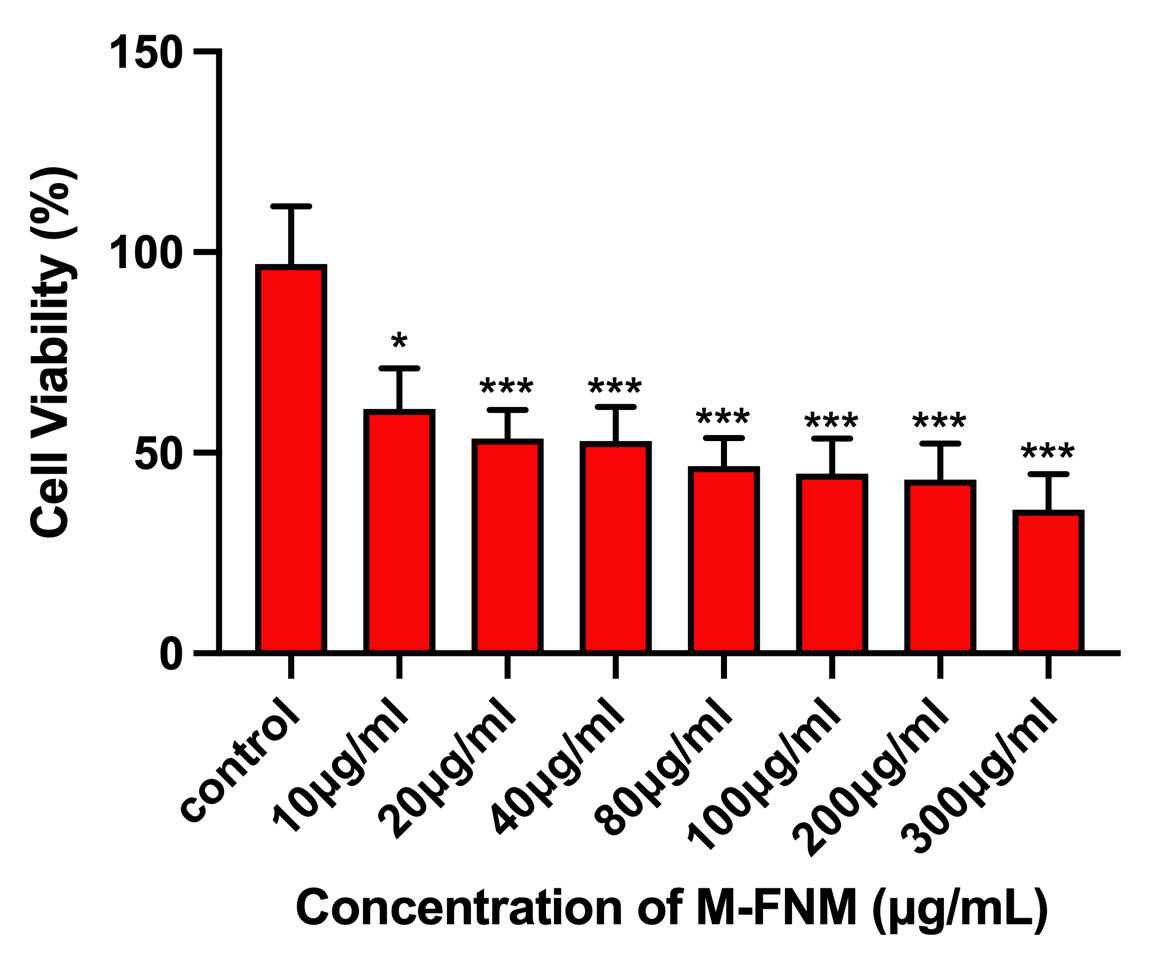
**

**Supplementary Figure S6.** CCK-8 detects the inhibition of different concentrations of M-FNM on cell viability. Compared to control, **P* < 0.05, ***P* < 0.01, and ****P* < 0.001.

***Supplementary Figure S7***


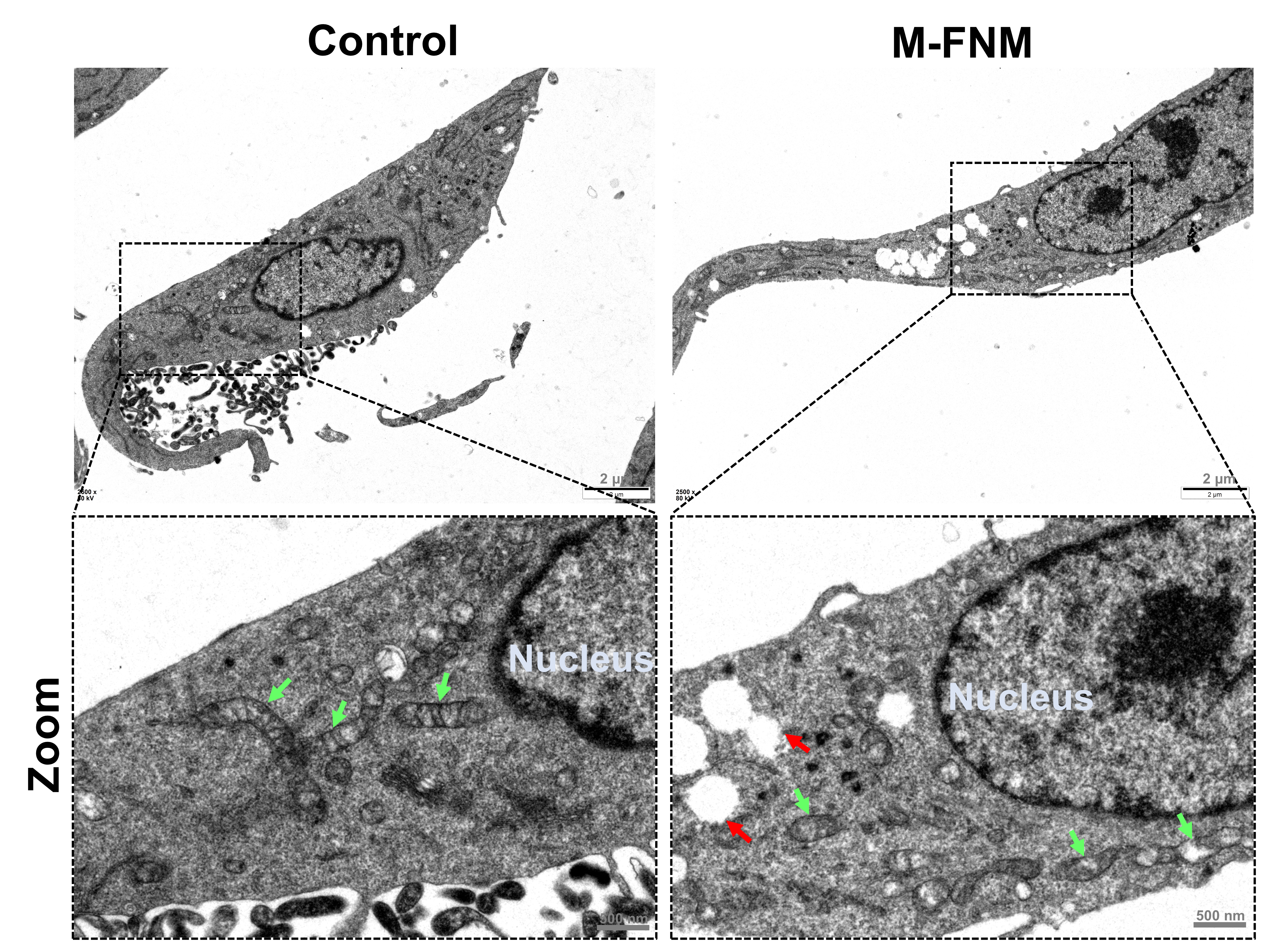


**Supplementary Figure S7.** TEM image of CAL27 cells upon M-FNM (300 μg/mL) treatment for 12 h. The green arrows indicate the mitochondria, while the red arrows symbolize the vacuole located within the cytoplasm.

***Supplementary Figure S8***

**

**

**Supplementary Figure S8.** ESR spectra of •OH captured by DMPO after M-FNM treatment.

***Supplementary Figure S9***

**
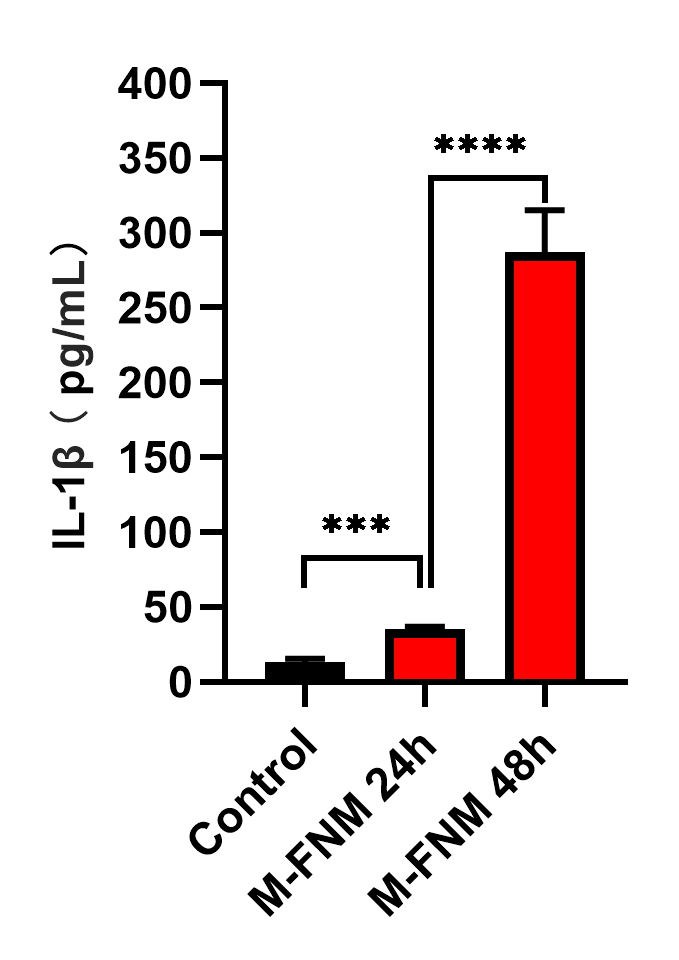
**

**Supplementary Figure S9.** IL-1β release in supernatant after M-FNM (300 μg/mL) treatment. Compared to control or M-FNM, **P* < 0.05, ***P* < 0.01, ****P* < 0.001, and *****P* < 0.0001.

***Supplementary Figure S10***

**
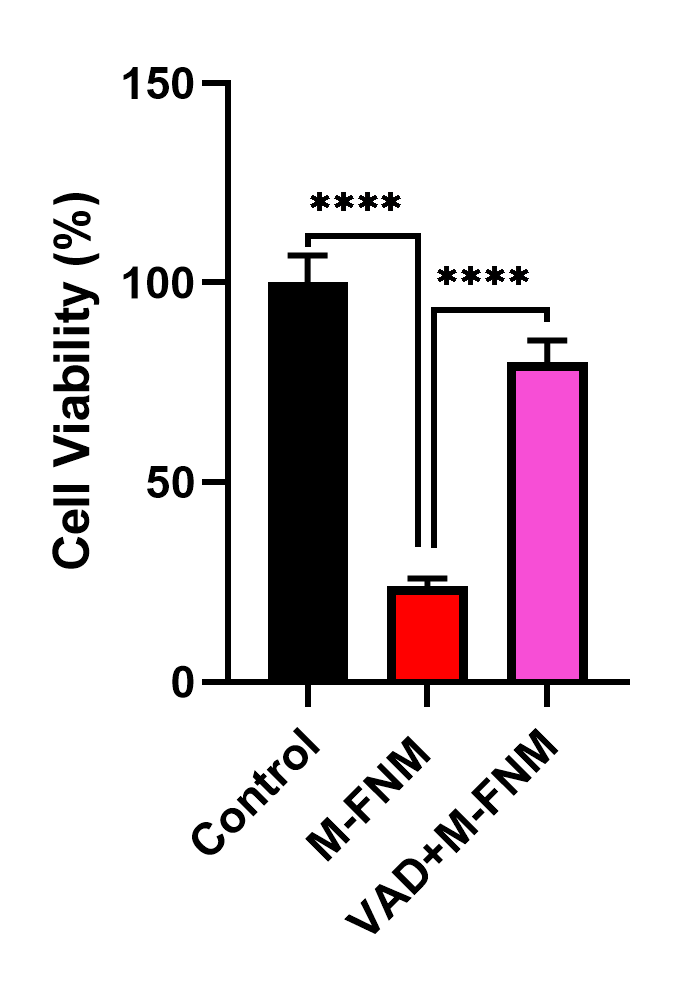
**

**Supplementary Figure S10.** Rescue of cell viability by Z-YVAD-FMK (VAD). CAL27 cells were pretreated with Z-YVAD-FMK (20 μM) for 1 h, then add M-FNM to continue co-cultivation for 24 h. Compared to control or M-FNM, **P* < 0.05, ***P* < 0.01, ****P* < 0.001, and *****P* < 0.0001.

***Supplementary Figure S11***

***
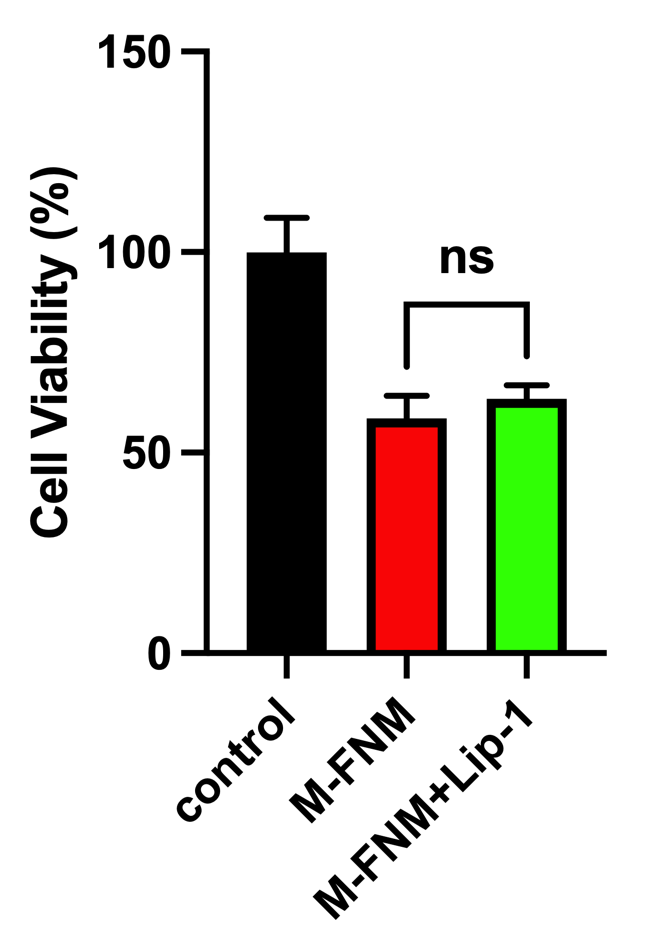
***

**Supplementary Figure S11.** M-FNM did not cause ferroptosis. CAL27 cells were pretreated with liproxstatin-1 (50 nM) for 12 h, then add M-FNM to continue co-cultivation for 24 h. Compared to M-FNM group, ns *P* > 0.05.

***Supplementary Figure S12***


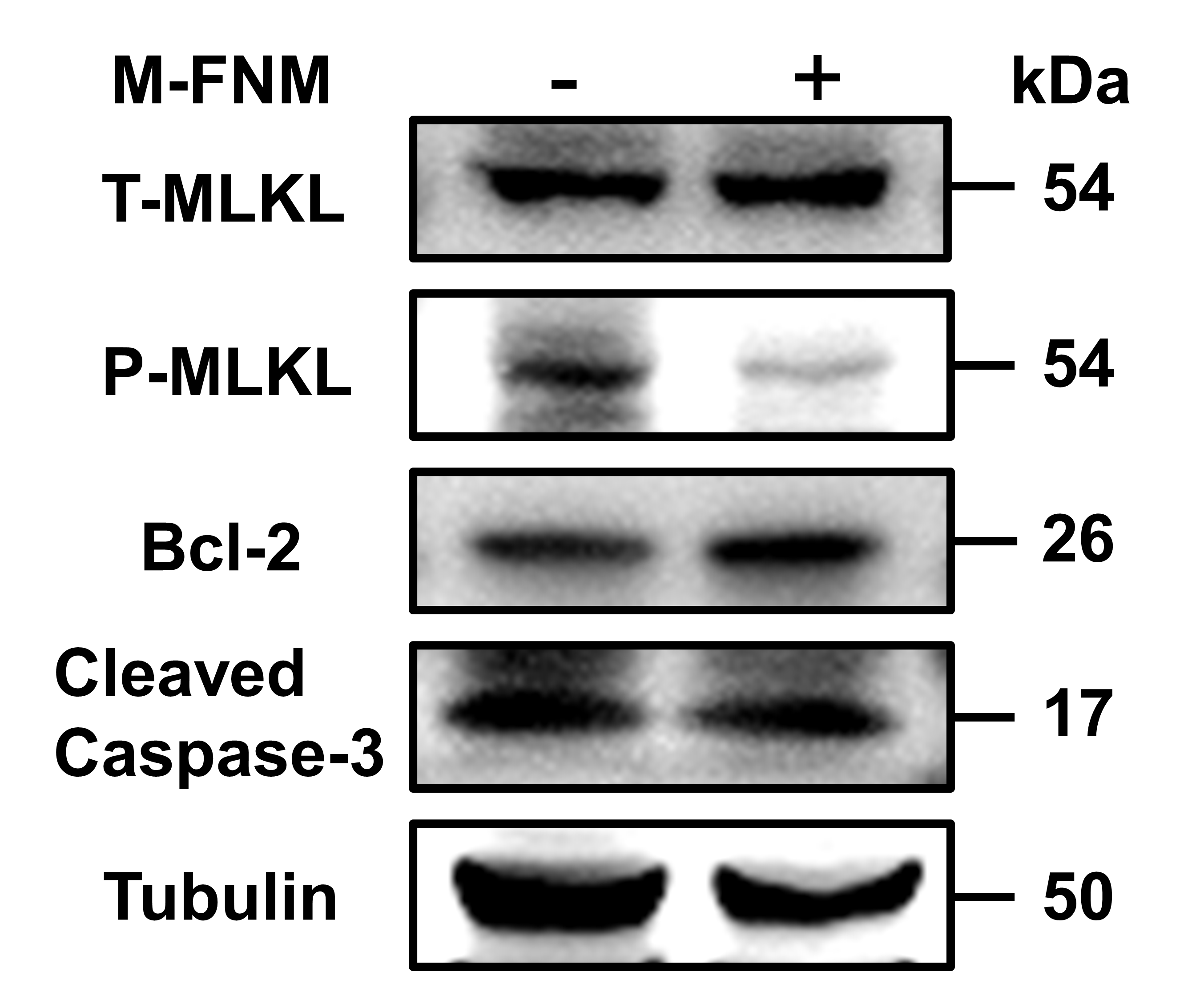


**Supplementary Figure S12.** M-FNM (300 μg/mL) did not influence expression of Total MLKL, phosphorylation MLKL, Bcl-2 and cleaved Caspase-3 by western blot.

***Supplementary Figure S13***

***
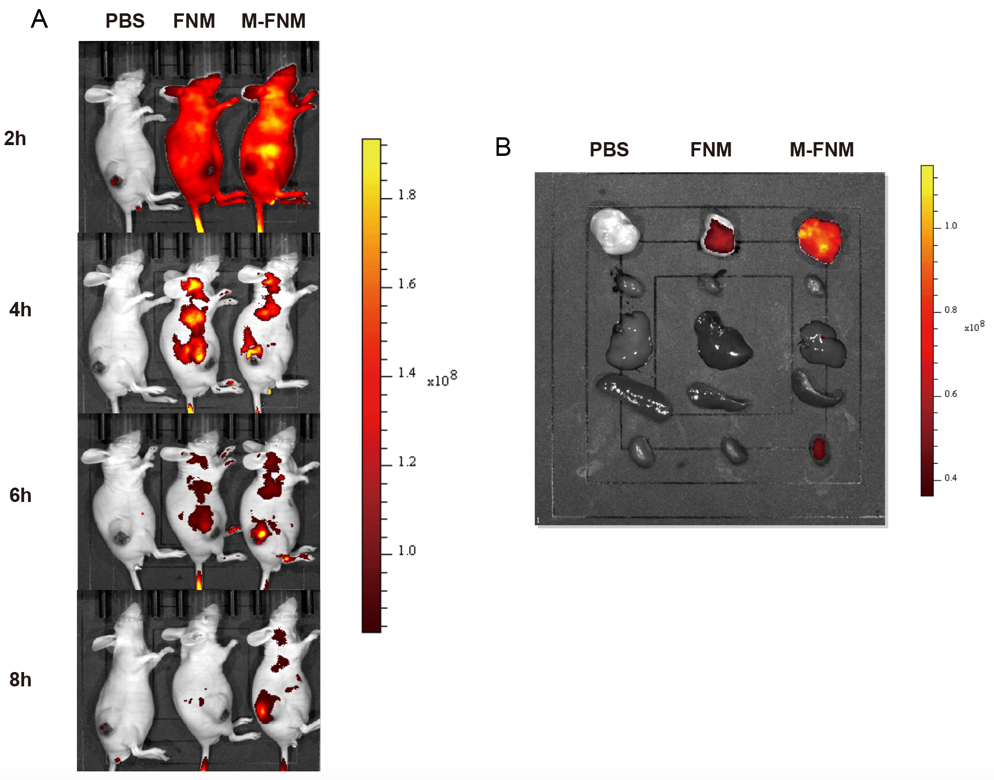
***

**Supplementary Figure S13.** Targeting effect and important organs distribution of M-FNM *in vivo*. The targeting effect of M-FNM compared with FNM in local tumors (A) and the recruitment of important organs (B).

***Supplementary Figure S14***


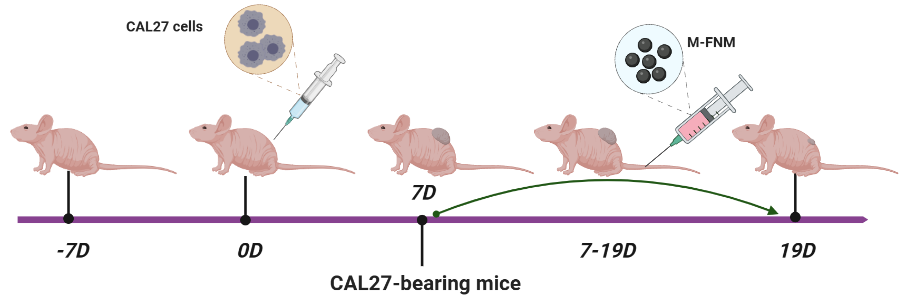


**Supplementary Figure S14.** Treatment schedule of utilizing M-FNM for antitumor therapy (BALB/c-nude, n=3).

***Supplementary Figure S15***

***
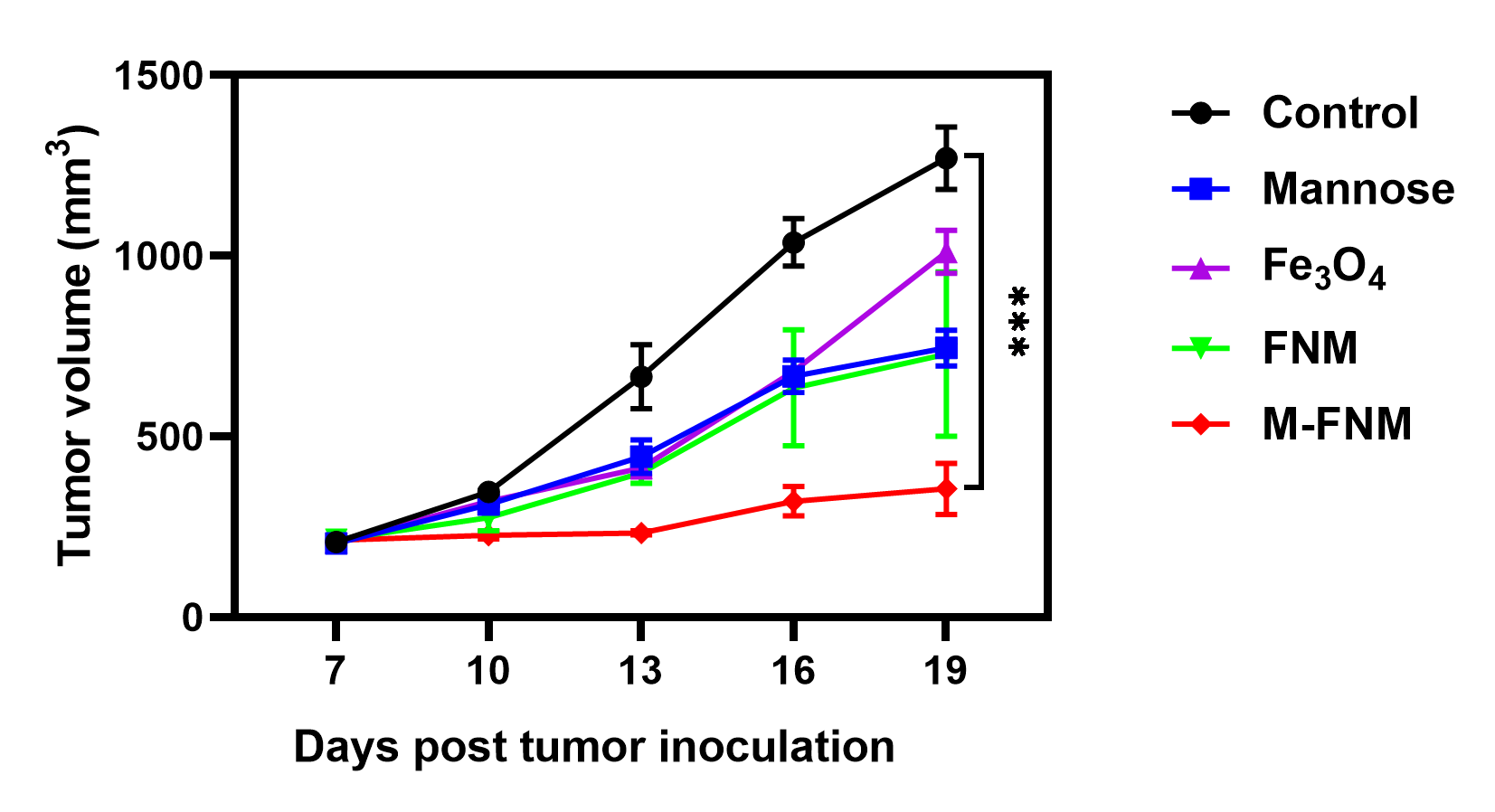
***

**Supplementary Figure S15.** Tumor volume of CAL27-tumor-bearing mice with different treatments (10mg/kg). Compared to control, **P* < 0.05, ***P* < 0.01, and ****P* < 0.001.

***Supplementary Figure S16***


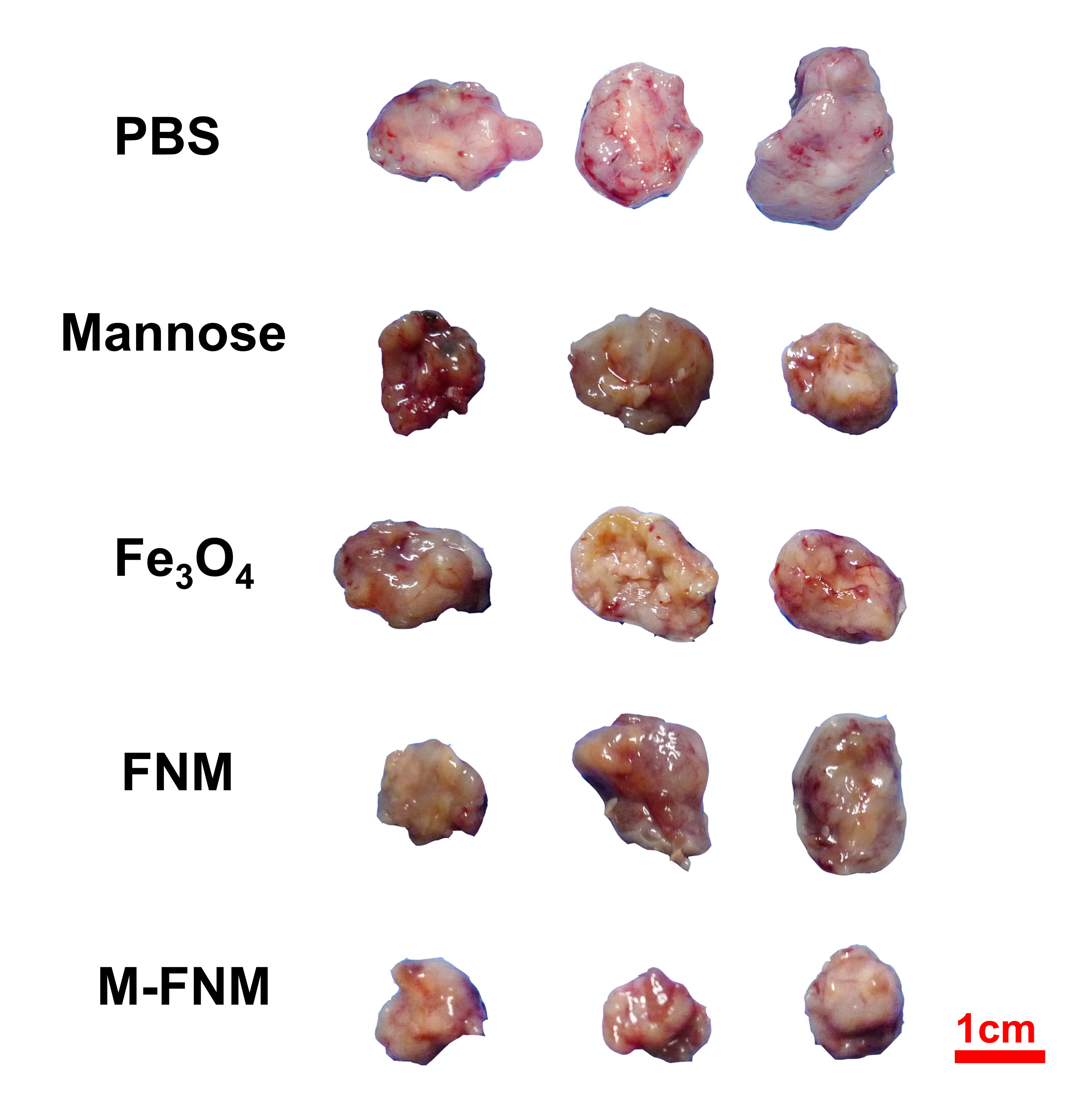


**Supplementary Figure S16.** Digital photos of excised tumors in different treatments.

***Supplementary Figure S17***

***
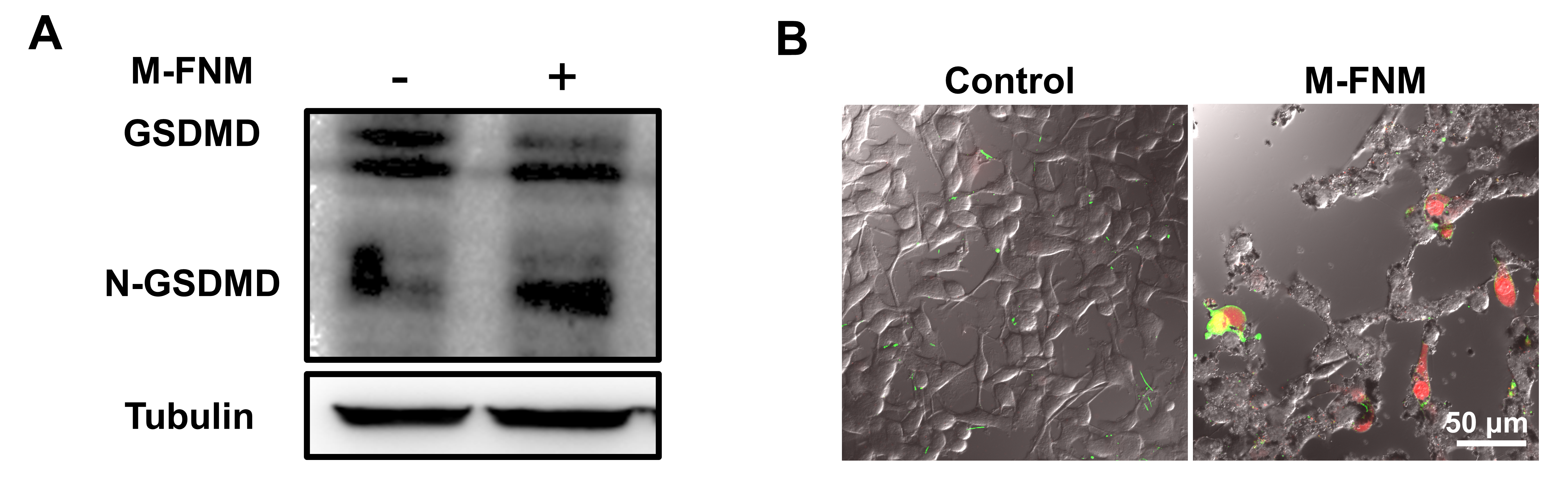
***

**Supplementary Figure S17.** SCC-7 cells undergo pyroptosis under the action of M-FNM. (A) M-FNM (300 μg/mL) promoted the expression of N-GSDMD in SCC-7 cells. (B) Annexin V/PI detected the damage of cell membrane integrity by M-FNM (300 μg/mL).

***Supplementary Figure S18***

**
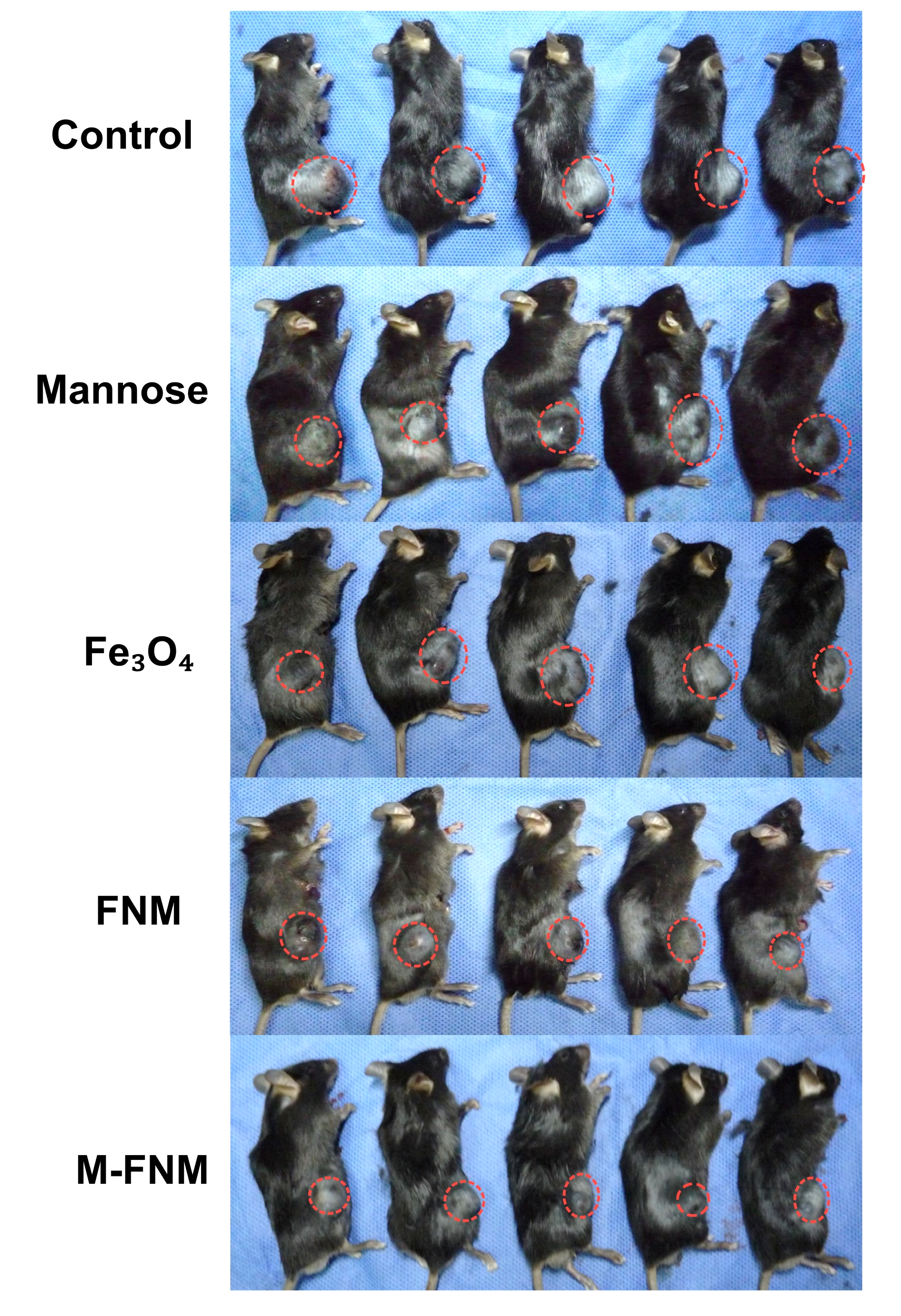
**

**Supplementary Figure S18.** representative photographs of SCC-7 tumor-bearing mice with different treatment.

***Supplementary Figure S19***


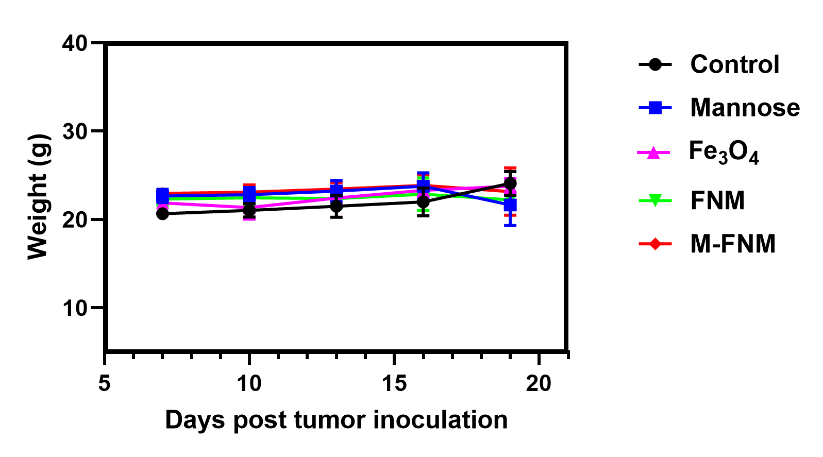


**Supplementary Figure S19.** Body weight of subcutaneous CAL27 tumor model in mice.

***Supplementary Figure S20***


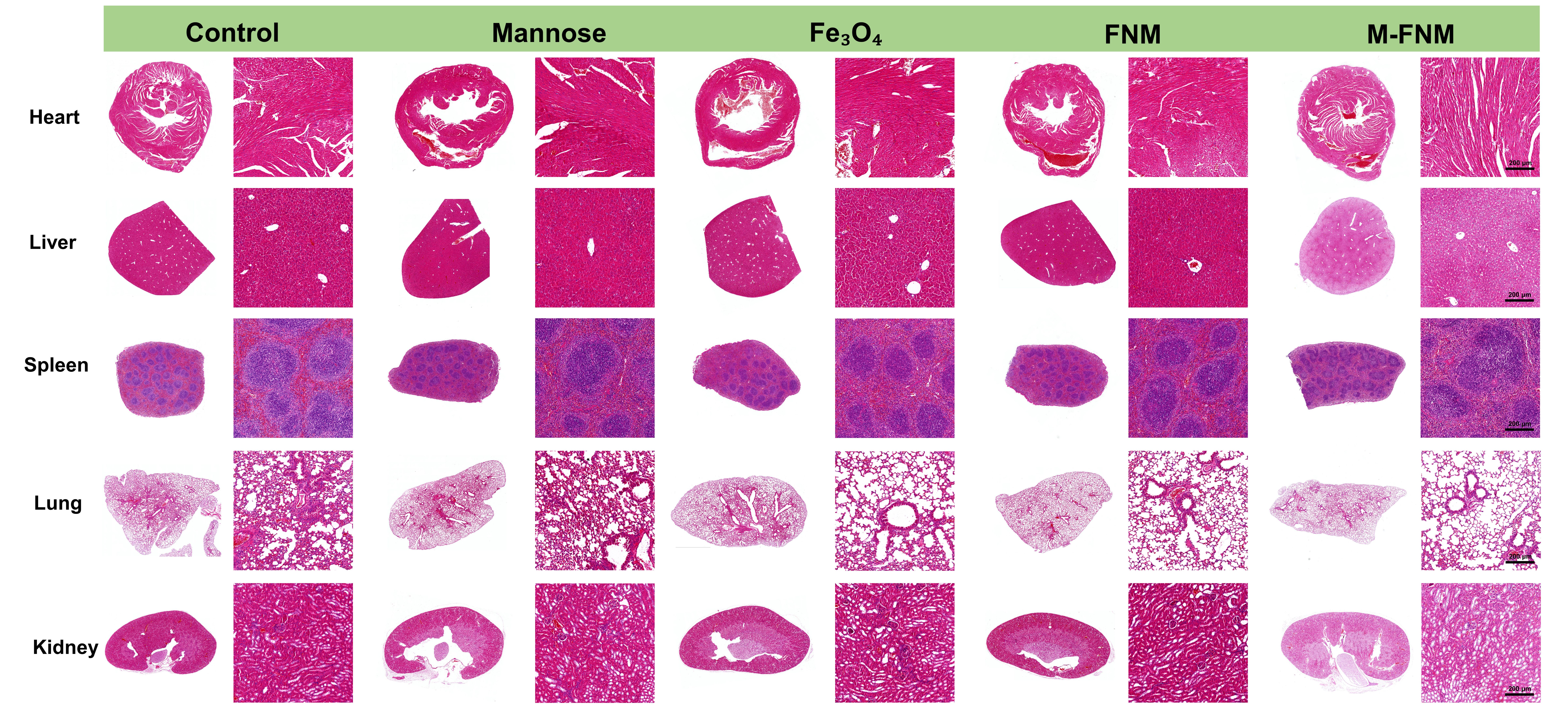


**Supplementary Figure S20.** H&E staining of organs. H&E staining images of the heart, liver, spleen, lung, and kidney from the mice in all groups.

***Supplementary Figure S21***


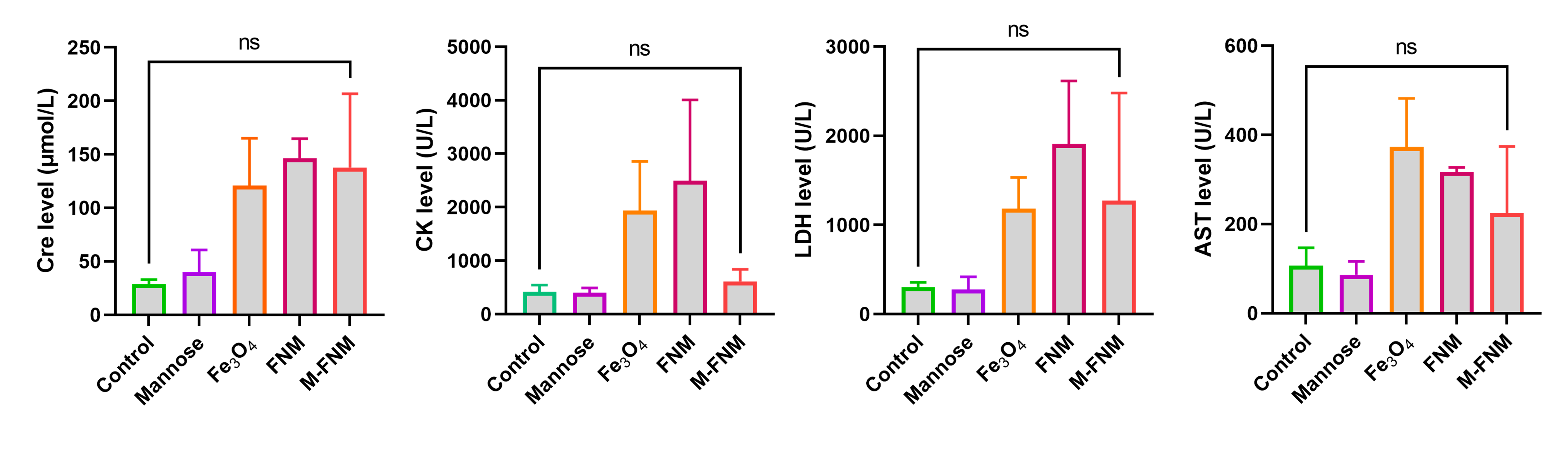


**Supplementary Figure S21.** Biosafety analysis of serum after treatment. Levels of creatinine (Cre), creatinine kinase (CK), lactic acid dehydrogenase (LDH) and aspartic acid transaminase (AST). Compared to control, ns *P* > 0.05.
